# Recent Advancements of LSPR Fiber-Optic Biosensing: Combination Methods, Structure, and Prospects

**DOI:** 10.3390/bios13030405

**Published:** 2023-03-20

**Authors:** Hongxin Zhang, Xue Zhou, Xuegang Li, Pengqi Gong, Yanan Zhang, Yong Zhao

**Affiliations:** 1 College of Information Science and Engineering, Northeastern University, Shenyang 110819, China; 2 The State Key Laboratory of Synthetical Automation for Process Industries, Shenyang 110819, China; 3 Hebei Key Laboratory of Micro-Nano Precision Optical Sensing and Measurement Technology, Qinhuangdao 066004, China

**Keywords:** LSPR, biosensor, label-free, optical fiber sensor

## Abstract

Fiber-optic biosensors based on localized surface plasmon resonance (LSPR) have the advantages of great biocompatibility, label-free, strong stability, and real-time monitoring of various analytes. LSPR fiber-optic biosensors have attracted extensive research attention in the fields of environmental science, clinical medicine, disease diagnosis, and food safety. The latest development of LSPR fiber-optic biosensors in recent years has focused on the detection of clinical disease markers and the detection of various toxic substances in the environment and the progress of new sensitization mechanisms in LSPR fiber-optic sensors. Therefore, this paper reviews the LSPR fiber-optic sensors from the aspects of working principle, structure, and application fields in biosensors. According to the structure, the sensor can be divided into three categories: traditional ordinary optical fiber, special shape optical fiber, and specialty optical fiber. The advantages and disadvantages of existing and future LSPR fiber-optic biosensors are discussed in detail. Additionally, the prospect of future development of fiber-optic biosensors based on LSPR is addressed.

## 1. Introduction

Biosensors of optic-fiber have been investigated and extensively applied in DNA quantitative detection [1,2], enzyme measurement [3], food quality detection [4], cancer biomarker detection [5], and pathogen [6] and toxic substances detection [7] in the environment.

There are some detection principles of fiber-optic biosensors, such as localized surface plasmon resonance (LSPR) [8], interference [9,10], whispering gallery mode (WGM) [11], surface plasmon resonance (SPR) [12,13], etc. Among them, biosensors based on LSPR are particularly attractive. LSPR occurs when the frequency of the incident light matches the collective oscillation frequency of the metal-free electrons [14]. The LSPR fiber-optic biosensor utilizes the inherent LSPR effect of metal nanoparticles to amplify the influence of the refractive index (RI) change of the surrounding environment on the resonance mode, thereby improving the sensitivity of the fiber-optic biosensor. Variations in the size and shape of metal nanoparticles (NPs) lead to exhibiting different properties making LSPR fiber-optic biosensing highly flexible [15]. In addition, it is also easy and cheap to immobilize noble metal nanoparticles on optical fibers. Moreover, the penetration depth of the evanescent field in the environment is only 100–200 nm [16], which makes the sensor only sensitive to changes in the environment near the surface of the optical fiber and greatly suppresses the influence of background noise in the environment. Furthermore, noble metal nanoparticles such as gold [17] and silver [18] have a certain biological affinity, so the sensor performance can be improved through the combined action of some biomolecules. In 2018, Luo et al. [19] used Staphylococcal protein A on the surface of gold nanoparticles to enhance the specific binding ability of anti-NDV monoclonal antibodies to NDV. It is precisely because of these characteristics of LSPR fiber-optic biosensors that LSPR fiber-optic biosensors have important research significance and application value in medical diagnosis, drug development, cancer detection, and other fields. Therefore, the combination of LSPR technology and fiber-optic sensing technology is an inevitable trend in the development of biosensing technology.

**Figure 1:**
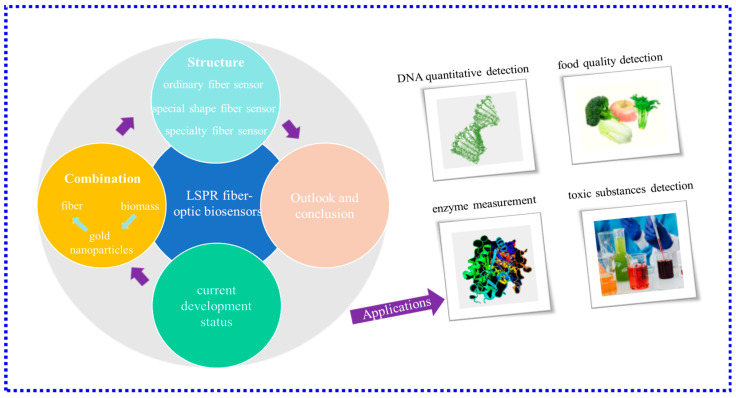
Schematic diagram of the main content of this review.

With continuous improvement and development in the field of materials and advanced optical fiber manufacturing technology, some unique probes that utilize the LSPR effect have sprung up. This paper reviews the latest progress of LSPR fiber-optic biosensors, especially the innovative progress in structure and its application in practical scenarios in the past two years, as well as the new sensitization methods in recent years. Figure 1 shows the main content of this review. The advantages and disadvantages of LSPR fiber-optic biosensors in recent years are summarized, and their further development directions are prospected.

## 2. Sensing Principle and Combination

### 2.1. Ways of Optical Fiber Generate LSPR

**Figure 2:**
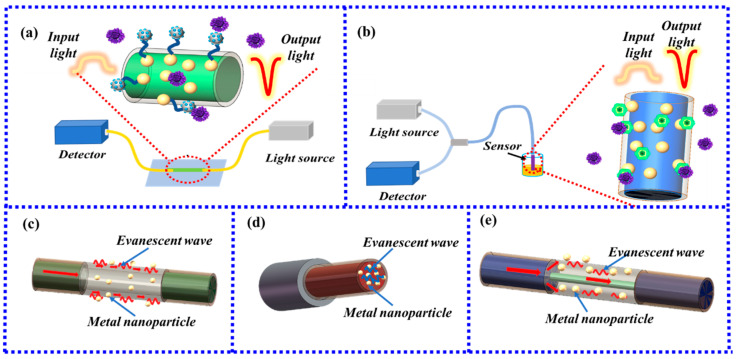
(**a**) Transmissive structure; (**b**) Reflective structure; (**c**) Hollow core fiber LSPR sensor; (**d**) Fiber end face LSPR sensor; (**e**) Fiber core mismatch LSPR sensor.

There are two types of measurement structures of LSPR fiber-optic sensors, one is the transmission structure [20], and the other is the reflection structure [21]. The transmissive structure is mainly connected to the light source at one end of the sensor and the signal-receiving device at the other end. The fiber-optic sensing area in the middle excites the metal nanoparticles through the evanescent field generated by the optical fiber to generate LSPR to achieve sensing performance. The transmissive structure is shown in Figure 2a. The reflective structure uses one end of the sensor to transmit the light source signal and also receive the sensing signal through the fiber-optic coupler. The other end of the sensor is coated with metal nanoparticles to generate LSPR for sensing. The reflective structure is shown in Figure 2b.

When the incident light enters the optical fiber, the evanescent field excites the gold nanoparticles producing the LSPR phenomenon. The LSPR peak, denoted by *ω_max_*, is as follows:

(1) $$\omega_{max}=\frac{w_{p}}{{\left(2\varepsilon_{m}+1\right)}^{1/2}}$$

*λ* = 2πc/*w* and *ε_m_* = *n*^2^, Formula (1) can be changed to:

(2) $$\lambda_{max}=\lambda_{p}\sqrt{2n_{m}^{2}+1}$$

where *λ_max_* is the LSPR peak wavelength and *λ_p_* is the wavelength corresponding to the plasmon frequency of the bulk metal. From Formula (2), it can be known that there is a linear relationship between the LSPR peak wavelength and the RI of the surrounding medium. When biomolecules bind to metal nanoparticles and the interaction between biomolecules and biomolecules is adsorbed on metal nanoparticles, the resonant absorption peak changes with the surrounding RI changes due to changes in the RI of the local environment [22].

Both structures need to utilize the evanescent field generated by the fiber to excite the LSPR effect of metal nanoparticles for sensing. There are three ways to generate the evanescent field of an optical fiber. One is to use the evanescent field generated by the fiber itself. This kind of fiber has a thin cladding or no cladding structure in the sensing area, so the evanescent field generated by the fiber can be conducted to the metal nanoparticles to excite LSPR to realize sensing, such as hollow core fiber (HCF), microstructured optical fiber (MOF), or the fiber end face, etc. The outer surface of a common HCF is coated with metal nanoparticles to excite LSPR, as shown in Figure 2c, and the LSPR sensing structure on the fiber end face is shown in Figure 2d. The second type is to make certain structural changes to the fiber so that the evanescent field can penetrate to the surface of the fiber, such as multi-core fiber (MCF) or photosensitive fiber (PSF) treated with chemical reagents, reducing the thickness of the cladding, changing the shape of the fiber probe. Common special-shape fibers include tapered fiber, U-type fiber, Ω-type fiber, D-type fiber, and S-type fiber. The third type is to use the core mismatch structure. The core mismatch is to use the difference in the core diameter of different optical fibers so that part of the light in the fiber with a large core diameter enters the cladding of the fiber with a small core diameter for transmission. The evanescent field is generated at the interface between the fiber cladding with a small core diameter and the environment, and the metal nanoparticles on the fiber surface are excited to generate LSPR to realize sensing. The common SMF-MMF-SMF structure is shown in Figure 2e.

### 2.2. Ways of Biomass Binding to Gold Nanoparticles

At present, there are many kinds of biomass detected by fiber-optic biosensors, such as protein [23], hormones [24], and DNA [25]. The recognition method of the target detection substance is usually specific recognition, specific bacteria recognition, and physical adsorption of mesopore size, as shown in Table 1.

### 2.3. Combination of Gold Nanoparticles to Optical Fiber

#### 2.3.1. Chemical Bond Method

LSPR is an optical property of noble metal nanoparticles, but AgNPs [31] and CuNPs [32] are easily oxidized by air. AuNPs have been widely used in experiments due to their good structural stability, so in this review, the binding method of gold nanoparticles and optical fibers is mainly explained. At present, the most common binding method is to fix gold nanoparticles to the surface of the optical fiber by chemical bonding, which is low-cost and simple to operate. The most commonly used methods are the silane method and the electrostatic self-assembly method [33] (also known as the polyelectrolyte (PE) assembly [34] method).

**Figure 3:**
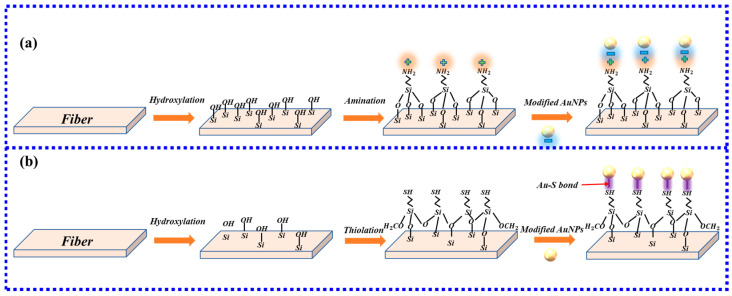
(**a**) aminosilanization process; (**b**) mercaptosilane modified method’s process.

At present, gold nanoparticles are immobilized on fiber-optic biosensors mainly by the silane method [35]. Silane methods include aminosilanization [36] and mercaptosilane modified method [37]. The principle of aminosilanization is positive and negative binding. The most common aminosilanization is to use a solution such as (3-Aminopropyl)triethoxysilane (APTES) [38] or (3-Aminopropyl)trimethoxysilane (APTMS) [39] for amination. The aminated fibers are positively charged, and the gold nanoparticles are negatively charged [35]. Using the electrostatic force between the two, that is, the combination of ionic bonds, the gold nanoparticles are fixed on the fiber, as shown in Figure 3a. Mercaptosilane modified method mainly uses (3-mercaptopropyl)trimethoxysilane [40] (MPTMS) to modify optical fiber. AuNPs are attached to the SH group [41] of MPTMS, which helps in the formation of a thiol-terminated self-assembled monolayer (SAM) of MPTMS over optical fiber, that is, Au-S covalent bonding [42]. MPTMS has methoxy groups (Si-OCH3), which can be hydrolyzed to silanol (Si-OH) groups. The Si-OCH3 interacts with the partially methoxy-hydrolyzed silanol [43] to form a self-assembled film with thiols end groups on the surface of the fiber, as shown in Figure 3b.

The silane method requires a long immersion in the solution for each step in the functionalization process, which increases the experimental time to a certain extent, thus limiting the application of LSPR sensors.

The electrostatic self-assembly method has a fast electrostatic adsorption speed and significantly shortens the deposition time [44], which solves the shortcomings of the silane method. The electrostatic self-assembly method mainly uses colloids with different charges to realize the rapid connection between optical fibers and gold nanoparticles. The fiber, after hydroxylation, is negatively charged. The gold nanoparticles prepared by the chemical reduction method also have negative charges, so it is necessary to coat the surface of the fiber with the cationic polyelectrolyte to make the surface of the fiber with a positive charge. Commonly used cationic polyelectrolytes are poly(allylamine hydrochloride) (PAH) and poly(diallyldimethylammonium chloride) (PDDA). Common anionic polyelectrolytes are poly(styrene sulfonate) (PSS) and poly(acrylic acid, sodium salt) (PAA). Moreover, the structure of the polyelectrolyte, which can avoid the aggregation of nanoparticles to a certain extent [45], is of great help in improving the performance of the sensor.

**Figure 4:**
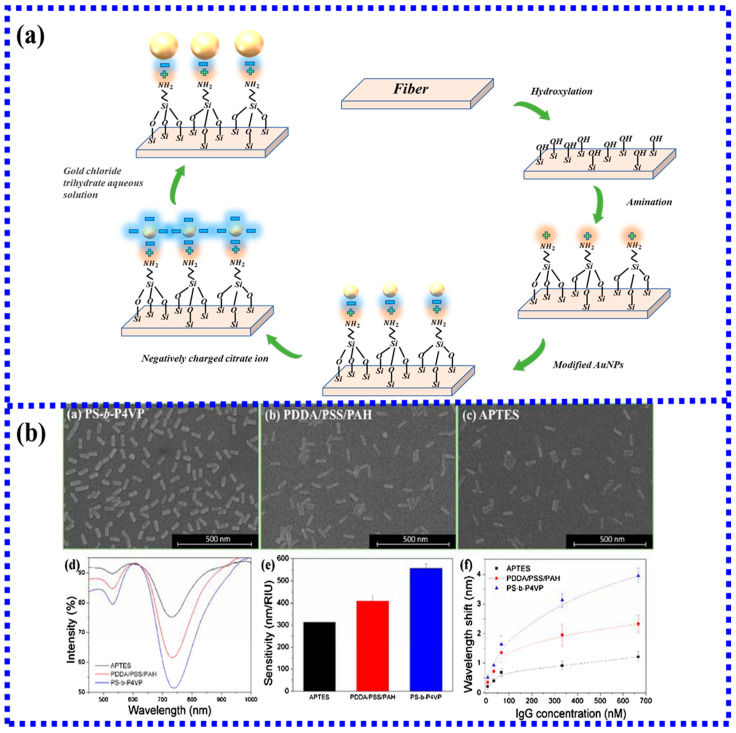
(**a**) The method of increasing the volume of gold nanoparticles by the secondary immersion in citrate; (**b**) Comparison of the BCP method with traditional electrostatic self-assembly and silane method [48]. Copyright 2023, Elsevier.

The silane method and the electrostatic self-assembly method described above are both methods of particle self-assembly. Although this method is simple to operate, it will cause phenomena such as aggregation [46], which will reduce the sensitivity of the sensor. Therefore, in recent years some new methods have been proposed to improve the traditional self-assembly method. The first very innovative method is the secondary growth method of gold nanoparticles proposed by Hyeong-Min Kim [47] in 2019. The traditional aminosilanization method was used to grow gold nanoparticles on the fiber. Then the citrate secondary immersion method was used to increase the volume of the gold nanoparticles, which solved the limitation of the volume of gold nanoparticles by traditional methods and improved the stability of nanoparticles. The functionalization is shown in Figure 4a.

In 2021, a new method using spherical gold nanoparticles [46] and gold nanorods [48] to prepare self-assembly templates by block copolymer (BCP) and surfactant modification further improved the electrostatic self-assembly method. The cationic surfactant CTAB was used in the AuNR growth solution. The new method to remove the CTAB by three cycles of PSS treatment was highly efficient, as was the subsequent displacement of PSS by sodium citrate. To better reflect the advantages of this new method, another two sensors were fabricated simultaneously using two conventional self-assembly methods. By comparison, it is found that the sensor prepared by the block copolymer method is far superior to the traditional method in terms of particle distribution on the sensor surface, sensor sensitivity, and specific detection performance. The results are shown in Figure 4b.

The LSPR fiber-optic sensor based on chemical bonding has poor reproducibility due to the lack of a fixed position of the nanoparticle distribution and the uncertainty of the number of nanoparticles in each coating process. Because of the weak chemical bond adhesion, the bonding between the nanoparticles and the optical fiber is not strong. So it is easy for some nanoparticles to fall off the surface of the fiber during the functionalization process [47], resulting in a decrease in the repeatability and durability of the sensor. Moreover, during the measurement process, the nanoparticles will aggregate on the surface of the fiber [49] due to the surface tension of the sensor surface.

#### 2.3.2. Non-Chemical Bond Method

To maintain the stability of the sensor for a long time, traditional physical methods are used to prepare sensors with neatly arranged nanoparticles and a constant number of nanoparticles, such as focused ion beam (FIB) milling [50], electron beam-induced deposition [51] (EBID), and electron beam lithography (EBL) [52]. Besides, new methods such as microelectromechanical systems [53] (MEMS) technology are also proposed to prepare sensors with neatly arranged nanoparticles and a constant number of nanoparticles.

**Figure 5:**
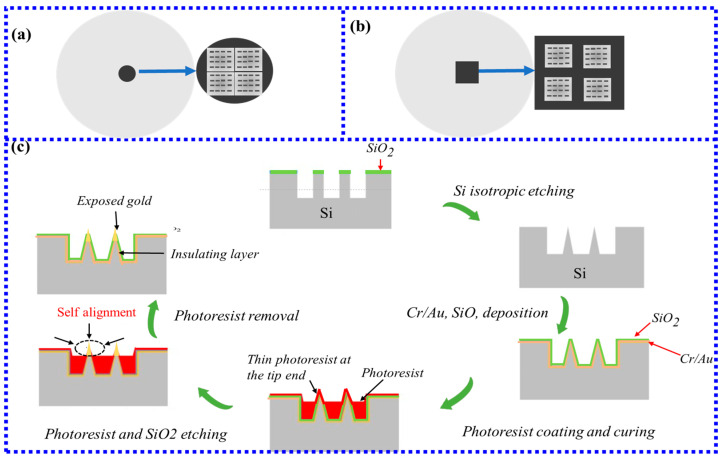
(**a**) Periodic arrays of FIB fabricated gold nanopillars on the cleaved end-faces of four-mode optical fibers; (**b**) Arrays of square nanopillars on the tip of a multimode optical fiber. (**c**)The fabrication process of the microtip-array-based LSPR sensor [53]. Copyright 2023, Elsevier.

The FIB milling method mainly applies focused ion beam milling to fabricate nanostructures on the end face of the optical fiber deposited with a 40–100 nm thick gold layer. Anuj Dhawan’s team [54] first used the FIB milling method to prepare square and elliptical nanopillar arrays at the end face of F-MLD multimode fiber and conducted FDTD analysis on plasma nanostructures, as shown in Figure 5a,b. The experimental results demonstrate that the gold nanodot array on the fiber tip can be used for sensing probes for chemical and biological analytes while providing a new solution to make LSPR fiber-optic biosensors. However, the FIB milling method on the fiber surface first proposed took a long time and was expensive. In 2019, the Hyeong-Min Kim team improved [55] the FIB milling method on the end face of MMF, ensuring that the overall process time is minimized while maintaining the quality of the nanopattern.

EBID method is a vapor-phase direct-writing technique with sub-10 nm resolution that can be applied to micro- and nano-scale object manipulation [56]. EBID method does not require pre-deposition of resist on the substrate. Chemical precursors are physically adsorbed onto the substrate from the gas phase, and incident electrons stimulate their decomposition into volatile species, which are pumped away, leaving a non-volatile deposit behind. Using a tightly focused electron beam, nanoscale-shaped three-dimensional structures can be created [57]. In general, the preparation of nanoparticle patterns on optical fibers is usually realized by the combination of the EBID method and FIB milling method, which is called focused electron beam-induced deposition [58] (FEBID). In the FEBID method, the electron beam provides a smaller spot for better focusing and resolution, which is beneficial to the FIB milling method while avoiding FIB-induced gallium contamination in and near the deposited material [59].

Electron beam lithography (EBL) is a method of producing stamps for nanoimprint lithography. The targeted nanohole array pattern is transferred onto the fiber-optic mask by directing the electron beam to scan as it scans the resist-coated substrate. This way is more tightly than light and produces finer patterns, with a resolution below 10 nanometers. Professor Cusano’s team [60] used the EBL method to fabricate a metal-dielectric nanostructure grating on the end face of a single-mode fiber to achieve real-time dose monitoring, laying the foundation for future radiation monitoring in high-energy physics experiments.

A new method using a microtip array-based LSPR sensor was proposed in 2018 [53]. This method first prepared the tip by etching on the fiber end face, then deposited gold film and silicon material, and finally coated the bottom of the tip with a thin photoresist film, leaving only a small amount of bare gold nanoarrays at the tip to excite LSPR for sensing, as shown in Figure 5c. The RI sensitivities of the sensors with exposed gold lengths of 0.9 and 1.4 um were 8.96 RIU^−1^ and 9.94 RIU^−1^, respectively, which indicates that the new approach can make the sensor sensitive to changes in the surrounding medium without significantly reducing the contact area.

To sum up, the chemical bonding method and the non-chemical bonding method have their advantages and disadvantages. The chemical bonding method has a simple preparation process and low cost. However, because it is difficult to control the binding process of nanoparticles during the preparation process, gold nanoparticle shedding and nanoparticle aggregation are prone to occur. These phenomena will affect the performance of the sensor. The non-chemical bonding method has a controllable preparation process, so it has a compact structure, a small volume, a controllable number of nano-patterns, and a stable structure. However, the non-chemical bonding method requires the use of some advanced microfabrication techniques, which increases the manufacturing cost. The diversification of the combination of gold nanoparticles and optical fibers and the continuous development of manufacturing technology has created infinite possibilities for the fabrication of LSPR fiber-optic biosensors with various structures.

## 3. Structure Classification and Application of LSPR Fiber-Optic Biosensors

Due to the various ways gold nanoparticles bind to the optical fibers and the development of optical fiber fabrication technologies, a variety of LSPR fiber-optic sensors have been proposed. This section will be classified according to the shape of the fiber probe and the type of fiber, divided into three categories: ordinary fiber, special shape fiber, and specialty fiber.

### 3.1. LSPR Biosensors Based on Ordinary Optical Fibers

Ordinary optical fibers mainly refer to traditional SMF and MMF, which are widely used due to their mature manufacturing processes. Traditional optical fibers are usually made of silica and consist of a solid core surrounded by the cladding material with a slightly lower RI than the core [61]. If the angle of incidence is less than the critical angle, the incident light can be confined in the core region of the conventional optical fiber to achieve light propagation [62]. However, due to the large thickness of the cladding when it is not treated, the evanescent field cannot penetrate the cladding to excite the metal nanoparticles on the surface of the fiber to generate LSPR. Therefore, this type of sensor mainly realizes sensing by using the methods of core mismatch, reducing the thickness of the cladding, or using the fiber end face.

**Figure 6:**
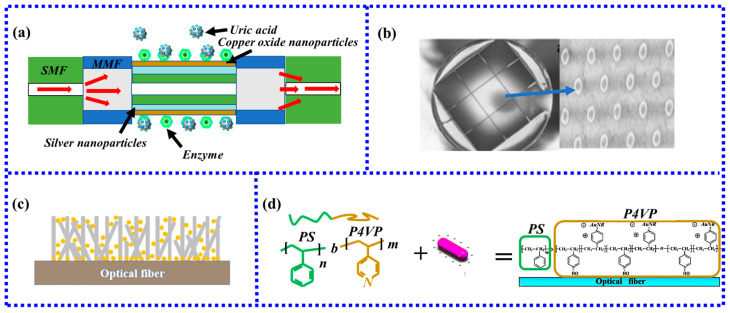
(**a**) Fiber-optic sensing probe for uric acid detection. (**b**) Gold nanodisk array sensing probe [64]. (**c**) Schematic diagram of ZnO nanowires and AuNPs composite structure [66]. Copyright 2023, Elsevier; (**d**) Schematic diagram of the PS-b-P4VP-templated citrate-AuNR monolayer [48]. Copyright 2023, Elsevier.

In a uric acid detection study, the SMF-MMF-SMF-MMF-SMF structure was combined with CuO-NPs and AgNPs and modified with uricase [63]. The particle size of the synthesized AgNPs in this study was 7.5 ± 0.5 nm, with a fixed point for the enzyme. The AuNPs were well distributed on the fiber, and the schematic diagram of the fiber structure is shown in Figure 6a. When the sensor is immersed in a solution containing uric acid, uricase in the solution interacts specifically with uric acid. The sensitivity of the sensor to measure uric acid is 6.15 nm/mM, and the detection limit is 69.26 μM. Therefore, the analysis of the performance of the proposed sensor shows that the LSPR-based fiber-optic biosensor can realize uric acid detection in a simple, fast, and low-cost manner.

The fiber end face method only coated the AuNPs at the end of the fiber. The mechanism of this method is that when the light is transmitted to the end of the fiber, it is reflected, and the evanescent field is generated to excite the LSPR phenomenon for sensing. Mollye Sanders et al. [64] prepared an LSPR fiber-optic biosensor for the specific detection of prostate proteins: the gold nanodisk array fabricated by the metal stripping process was fixed on the end face of the fiber, and free prostate-specific antigen was used to detect prostate proteins. The optical fiber end face and the enlarged view are shown in Figure 6b. The RI sensitivity of this sensor was measured at 226 nm/RIU, comparable to common gold nanoparticle array-based LSPR sensors. Compared with the other two structures of LSPR sensors, the sensor can be used in a narrower space and has a wider application range. However, due to the reflection loss of the LSPR fiber reflective structure, the combination of nanoparticles and high-Q optical cavities is prevented. Professor Cusano’s team proposed a new technique to improve plasmonic probe and fiber-optic sensing structures with large radiation losses [65]. In this approach, the inclusion of the plasmonic probe within the fiber resonator converts the wavelength shift of the LSPR into a change in the intracavity loss. Plasmon-induced losses are automatically compensated by a feedback loop acting on the intracavity optical gain element and locking the cavity in its impedance-matching condition. It has laid a solid foundation for the application of localized surface plasmon resonance optical fiber sensing in the direction of the optical fiber laboratory.

The performance of biosensors largely depends on the number of binding sites for biomolecules. For LSPR fiber-optic biosensors, the more gold nanoparticles, the more binding sites can be provided. Therefore, how to coat more gold nanoparticles in a limited sensing area is the key to improving sensor performance. Usually, the increase method of gold nanoparticles is to increase the sensing area or improve the immobilization efficiency of gold nanoparticles. Increasing the sensing area generally increases the length of the optical fiber, but the longer the optical fiber length, the greater the energy loss in the evanescent field and the worse the sensing effect. Kim et al. [66] proposed a new idea to increase the number of gold nanoparticles from the perspective of three-dimensional space, as shown in Figure 6c. The sensor first uses ZnO nanowires at the end face of the multimode fiber to change the two-dimensional plane into a three-dimensional structure and then fixes gold nanoparticles on the surface of the nanowires to increase the number of gold nanoparticles. Compared with the traditional two-dimensional structure, this structure not only enlarges the sensing area but also greatly improves the RI sensitivity and prostate antigen-specific sensitivity performance. RI sensitivity and specificity detection limits were improved by 171% and 404%, respectively. In another study, Lu et al. [48] fabricated an innovative new self-assembled template method to fabricate LSPR biosensors. A novel self-assembled template was prepared by surfactant modification and layer-by-layer electrostatic self-assembly for human IgG detection, as shown in Figure 6d. The results showed that compared with the traditional preparation method, the sensitivity of the new method was increased by 1.5 times.

**Figure 7:**
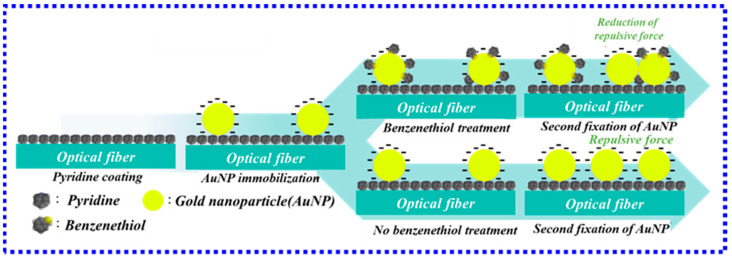
Schematic diagram of the fabrication process of fiber-optic LSPR sensors based on monomer (without benzenethiol treatment) and dimer (with benzenethiol treatment) [67]. Copyright 2023, Elsevier.

However, the small size of the nanoparticle sensing structure often leads to the inability of some large biomolecules to attach to the gold nanoparticles, resulting in a decrease in the sensitivity of measuring biomass. To solve this problem, the dimer structure was proposed to achieve biomass-specific detection and improve the performance of the sensor in 2021 [67]. The fabrication process of the monomer- and dimer-based fiber-optic LSPR sensors is shown in Figure 7. The sensor employed a dimeric longitudinal band-induced structure arranged at nanometer pitches to effectively increase the interaction of light with nanostructures [68]. Experimental results show that the longitudinal band shows an approximately 9.1-fold increase in sensitivity, and the dimer-based FO LSPR sensor also demonstrates an approximately 2.6-fold improvement in detection limit. In addition, molybdenum disulfide, graphene oxide, and other two-dimensional materials are used to improve the performance of LSPR biosensors because they can provide rich functional groups, have a large number of binding sites, high fixed density, good biocompatibility, and stability [69,70].

In recent years, LSPR fiber-optic sensors based on traditional single-mode and multimode fibers have achieved a lot of results in combination with new methods. Traditional optical fiber-based LSPR sensors have great potential in the field of biomass monitoring, but there are still urgent problems to be solved. First, the evanescent field is an important source to excite the LSPR effect, and the penetration depth of the evanescent field is only about 100–200 nm. Due to the limited geometry of traditional fibers, it is difficult for the evanescent field generated between the core and the cladding to penetrate the thickness of the cladding to excite LSPR for sensing. Second, the use of low-index/high-index materials in the core/cladding of the fiber is not possible [71] due to the light-guiding mechanism of total internal reflection. These two limiting factors limit the further improvement of the performance of LSPR-based fiber-optic biosensors.

### 3.2. LSPR Biosensors Based on Special Shape Fibers

**Figure 8:**
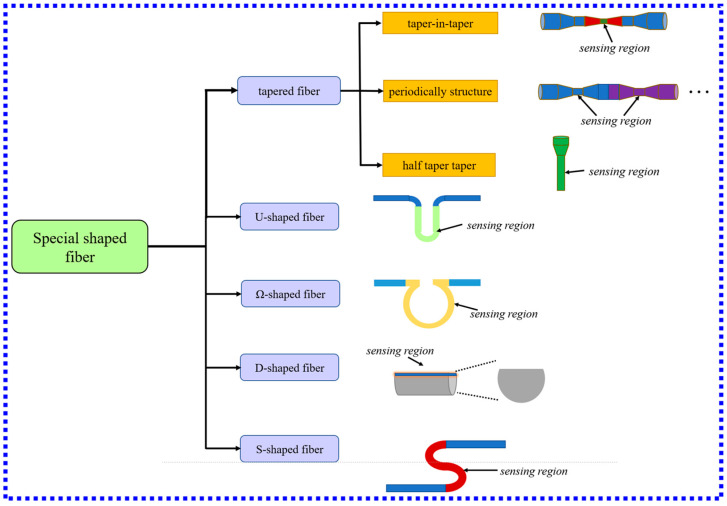
Schematic diagram of the classification and typical structure of LSPR biosensors with special shapes.

The special shape of the optical fiber and the structure of the specialty fiber make the LSPR fiber-optic biosensor well overcome these two limitations. These two kinds of fiber-optic sensors further improve the performance of the sensor and are well used in biomedical or clinical research. Therefore, improving the performance of LSPR fiber-optic biosensors by changing the shape and structure of the optical fiber has received extensive attention. LSPR fiber-optic sensors with special shapes are usually made of fibers into optical fibers with special shapes by tapering, heat treatment, and other methods to improve the performance of the sensor. According to different shapes, LSPR biosensors with special shapes can be roughly divided into several common shapes: tapered, U-type, Ω-type, S-type, and D-type. Figure 8 shows the classification of special shape LSPR biosensors and examples of typical structures under different classifications.

#### 3.2.1. LSPR Fiber-Optic Biosensor Based on Tapered Fiber

Similar to other types of LSPR sensors, tapering is the most basic strategy for changing the geometry of the sensor. Sensitivity, an important indicator of sensor performance, was reported to increase by a factor of 1.93 after tapering [72]. When the fiber is tapered, the reflected light in the tapered fiber core is reflected at an angle close to the critical angle, the penetration depth increases, and therefore the evanescent field in the cladding region increases. Within a certain range, as the diameter of the tapered fiber decreases, the evanescent field generated on the fiber surface is enhanced [73] so that the nanoparticles on the tapered fiber surface can be excited to generate LSPR.

**Figure 9:**
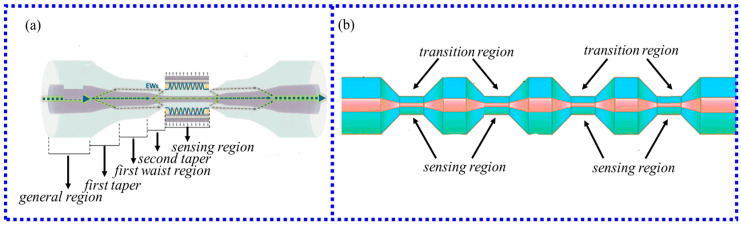
(**a**) Schematic diagram of taper-in-taper fiber structure [74]; (**b**) Schematic diagram of four periodic tapered fibers [75]. Copyright 2023, Elsevier.

In recent years, the structure of the tapered fiber has been continuously optimized, and the taper-in-taper fiber structure [74] and the periodically tapered structure [75] have appeared. In 2021, Zhi et al. [74] used the three-electrode semi-vacuum taper method to make the taper-in-taper fiber structure for the first time. The sensor used gold nanoparticles coated on the surface of the cone region to stimulate the LSPR effect and applied it to the detection of alanine aminotransferase. The structure is shown in Figure 9a; this structure further increases the taper processing operation based on the traditional tapered fiber structure to increase the penetration depth and strength of the evanescent field. The new structure also further improves the strength of the evanescent field and generates higher-power electron waves. The experimental results show that the sensor has a sensitivity of 4.1 pm/(U/L) and a detection limit of 10.61 U/L in the linear range of 10 to 1000 U/L, which has the potential to diagnose a liver injury.

Moreover, Zhu et al. [75] fabricated fiber structures with four/five/eight periodic tapers, as shown in Figure 9b. By comparing the sensitivity, ascorbic acid linear range, and detection limit of four/five/eight periodic tapered fiber sensors, the relationship between the number of tapered fibers and the sensing performance was explored. The experimental results show that the tapered fiber structure with five periods is superior to the four and eight-periodic tapered fiber sensors regardless of the linear range, the linear fitting degree, and the sensor sensitivity. It is an excellent choice for the effective detection of ascorbic acid in practical applications.

The structure of the tapered fiber is simple to operate, easy to manufacture, and has low manufacturing costs. Truncation to form a reflective probe structure has the advantage of being able to measure narrow, hazardous areas. However, after the tapered fiber is taper-drawn, it becomes more fragile and easier to break than the original fiber structure, which needs to be further overcome.

#### 3.2.2. LSPR Fiber-Optic Biosensor Based on U-Type Fiber

The bending sensing area of the U-type fiber sensor improves the sensitivity by changing the angle of the light perpendicular to the core-cladding interface. The RI sensitivity of U-type fiber is affected by the outer bending diameter of the U-type fiber probe. The sensitivity of the sensor increases as the bending radius of the probe decreases [76]. The sensitivity reaches a maximum when the diameter is reduced to a certain value and then decreases with the further reduction of the bending radius. Therefore, the sensitivity of the U-type fiber sensor can be improved by exploring its optimal bend radius.

**Figure 10:**
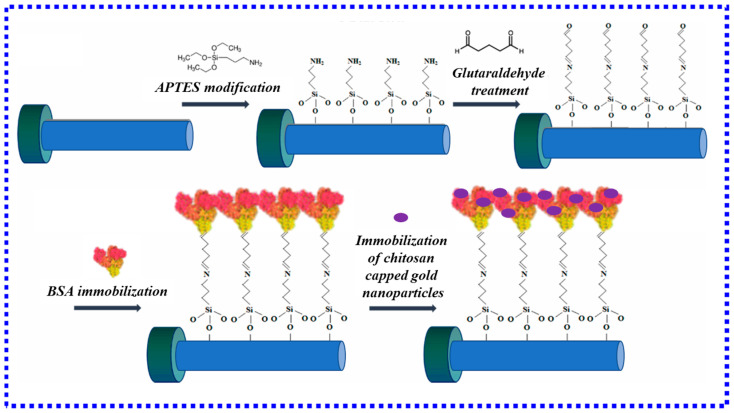
Chitosan-capped gold nanoparticles synthesized and immobilized on bovine serum albumin functionalized optical fiber [77]. Copyright 2023, Elsevier.

Combining the U-type fiber with other biological methods can not only achieve ultra-high sensitivity detection and biomass sensing performance but also use the interaction between biomass and heavy metal ions to detect heavy metal ions in the environment. In this regard, Kapil Sadani and colleagues [77] synthesized a chitosan-coated gold nanoparticle incorporated into a fiber-optic biosensor for qualitative and quantitative analysis of mercury ions in biological and environmental samples, as shown in Figure 10. The results showed that the total standard error of all samples was less than 15%, and the coefficient of variation was less than 12%, suitable for multiple purposes. This is the first mercury ion sensor suitable for diverse applications. In another study, PallaviHalkare et al. [78] proposed a method to detect Hg^2+^ and Cd^2+^ in water using Escherichia coli B40. Metal ions interact with thiols and other surface groups present on bacterial cells, resulting in a change in the refractive index around the AuNP-coated sensor probe, and Hg^2+^ and Cd^2+^ are specifically quantified in the linear range from 0.5 ppb to 2000 ppb, LOD is 0.5 ppb.

The U-type fiber-optic sensor is simple to prepare and can improve the sensing performance of the LSPR fiber-optic biosensor. Besides, the structure of the U-type sensor probe can also penetrate some narrow gaps. However, the fiber is easily broken during the bending process, so it is difficult to reduce the diameter of the U-type fiber probe to a certain diameter and cannot be further reduced.

#### 3.2.3. LSPR Fiber-Optic Biosensor Based on Ω-Type Fiber

Ω-type fiber can also improve the sensitivity of the sensor. The improvement in the performance of Ω-type fiber-optic biosensors is because the optical fiber will attenuate light in the bending part due to the interaction with the surrounding environment, resulting in bending loss [79]. The Ω-type fiber has a smaller radius of curvature and longer bending region length than the U-type fiber [80], so the RI sensitivity is higher. 

**Figure 11:**
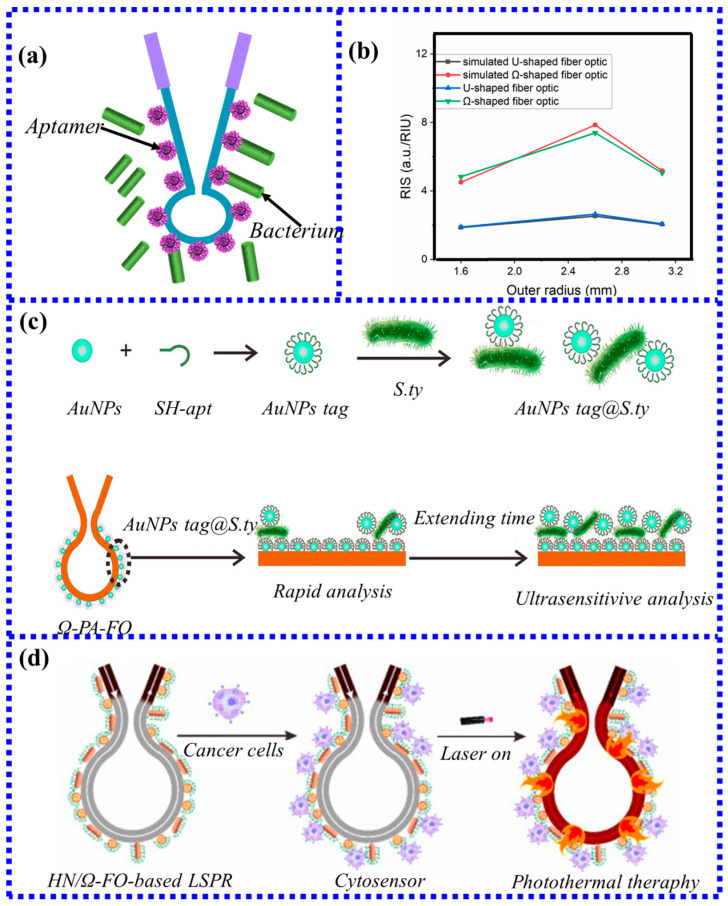
(**a**) Schematic diagram of Ω-type sensing probe; (**b**) The relationship between the outer diameter of U-type fiber and Ω-type fiber and the RI sensitivity [80]. Copyright 2023, Elsevier. (**c**) Schematic illustration of the Ω-FOLSPR for Salmonella typhimurium detection. AuNPs tag is the conjugation of the SH-apt and AuNPs [82]. Copyright 2023, Elsevier. (**d**) Hybridization and plasmonic photothermal treatment [83]. Copyright 2023, Elsevier.

Ω-based LSPR fiber-optic biosensors are highly sensitive, cost-effective, label-free, and miniaturized. Therefore, Ω-type LSPR fiber-optic biosensors are often used in cell screening and biological cell detection. Fluorescence experiments were used to demonstrate the experimental principle of cell binding, and the specific binding process could be clearly seen by binding the stained cells to the aptamers on the optical fiber for 2 h. In 2018, Luo et al. [81] proposed for the first time a new type of Ω-type LSPR fiber-optic biosensor combined with fluorescence experiments to achieve specific detection of Salmonella typhimurium. Figure 11a depicts a biosensor based on an Ω-shaped optical fiber for the detection of S. typhimurium (an important indicator for food safety inspection). The detection of S. typhimurium captured on the surface of a functionalized electrode with antibodies was measured by measuring the change in intensity. The amount is proportional to the antigen in the solution. The fabricated microfluidic sensor can detect the LOD of 128 CFU/Ml, and the linear range is 5 × 102 to 1 × 108 CFU/mL. In this experiment, the sensor was also used in the actual detection of chicken samples infected with Salmonella typhimurium; the experimental results showed that the recovery rate and relative standard deviation of the sensor varied from 85% to 123% and 6.5% to 8.3%, respectively. This indicates that the novel Ω-shaped LSPR biosensor based on the aptamer has good detection performance in food analysis and environmental monitoring.

In addition, Luo et al. [80] also used the same type of Ω-shaped fiber-optic biosensor to study the specificity of MCF-7 cancer cells. Here, the performance of U-shaped and Ω-shaped fiber-optic biosensors was analyzed using simulation (Figure 11b). Analyzing the coverage of AuNPs enabled the binding of more AuNPs, thereby increasing the number of MCF-7 cancer cells captured. Compared with other U-shaped fiber-optic cell sensors, the Ω-shaped fiber-based LSPR sensor achieves fast and ultrasensitive detection of cancer cells.

Recently, some new sensitization methods have also been used to improve the sensitivity of the Ω-type sensors. In 2022, gold nanoparticles of two sizes [82] were proposed to achieve specific detection of Salmonella typhimurium by the sandwich method. The small-sized gold nanoparticles of about 15 nm can be quickly modified to the surface of the fiber, which saves preparation time. Large-sized gold nanoparticles of around 47 nm were combined with Salmonella typhimurium to form a mixture, which was used for antibody detection while generating strong signal enhancement, as shown in Figure 11c. This sandwich method has the unique property of time-dependent sensitivity enhancement, showing an excellent linear relationship with bacterial concentration at different detection times. The detection time is short, and the detection line is high, but it can achieve rapid measurement. Extending the detection time can achieve ultra-sensitive detection. The detection limit of 100 min becomes 1/14 of the detection limit calculated in 10 min. Therefore, the rapidity and sensitivity requirements of detection can be met only by extending the analysis time.

Lu and colleagues [83] integrated cellular sensors with plasmonic photothermal processing for specific detection of MCF-7 cancer cells. The sensor solves the problems of low nanoparticle coverage and non-reusable sensors of LSPR fiber sensors by alternately arranging spherical nanoparticles and gold nanorods to form hybrid nanolayers. In addition, due to the high-efficiency, localized, and geometry-dependent heat distribution properties of the Ω-shaped fibers modified by hybrid nanolayers, the maximum temperature of the sensor under the action of laser light can reach 80 °C, which can specifically kill the particles captured on the surface of the sensor or has potential for surrounding cancer cells with minimal damage to the non-target cells. Figure 11d shows a hybrid nanolayer modified Ω-shaped fiber biosensor using different kinds of nanoparticles.

Compared with U-type optical fiber, the Ω-type fiber-optic biosensor can further improve the performance and sensitivity due to the increase of bending parts. However, the Ω-type fiber-optic sensor has more bending parts than the U-type sensor, so it is easier to break during the bending process, the structure is fragile, and the stability is poor. This is a problem that needs to be further solved in the current Ω-type fiber-optic biosensors.

#### 3.2.4. LSPR Fiber-Optic Biosensor Based on S-Type Fiber

Compared with U-type fiber, S-type fiber has higher sensitivity due to its smaller bending radius and longer bending length. According to the experimental conclusion of Shraddha K, the RI sensitivity of S-type fiber is about 1.5 times that of U-type fiber [84].

**Figure 12:**
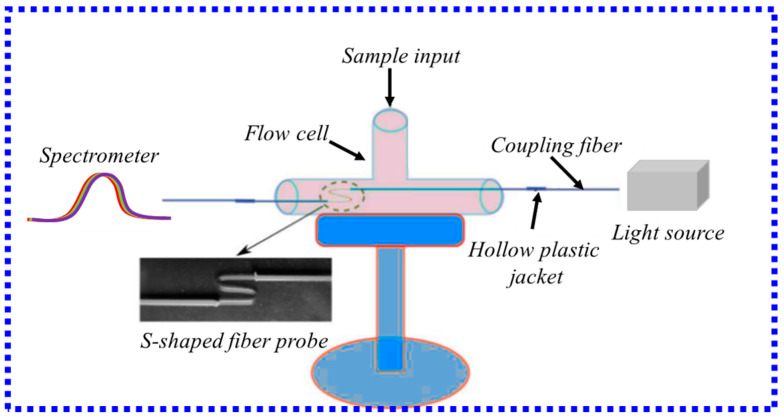
Schematic diagram of S-type optical fiber sensing device [85]. Copyright 2023, Elsevier.

In addition to being used to measure some chemical quantities, such as explosive traces, the S-type structure has also been applied to the measurement of biomolecules. In 2016, S. Chauhan et al. [85] proposed an S-type MMF optical biosensor to achieve the specific detection of goat anti-human immunoglobulin. The overall structure is shown in Figure 12. The transmission spectrum varies with concentration, with a detection limit of 1.7 nM for the sigmoid structure. The S-type biosensor has the advantages of small size and easy fabrication and can be widely used in disease screening and clinical diagnosis. Moreover, this highly sensitive S-type fiber probe can also further improve the sensitivity by using suitable noble metal nanoparticles to excite the LSPR effect on the probe surface.

Although the sensitivity of the S-type sensor probe is greatly improved compared with that of the U-type structure, due to its fragile structure in the preparation process, the S-type structure will be destroyed if the liquid flow speed is too fast. Therefore, at present, the experiment of realizing LSPR fiber-optic biosensors with S-type optical fiber is still less explored, and there is still much room for exploration. In the future, S-type LSPR fiber-optic sensors can improve the performance of optical fiber to increase the robustness of the structure.

#### 3.2.5. LSPR Fiber-Optic Biosensor Based on D-Type Fiber

The four kinds of optical fibers outlined above usually bend the optical fiber into different shapes without changing the structure of the optical fiber. The D-type optical fibers are mainly prepared by removing part of the cladding or removing the cladding and part of the core structure [86]. D-type fiber-optic sensors are easy to fabricate, and large evanescent waves can be obtained by removing part of the fiber, facilitating interaction with analytes. Additionally, the D-type fiber provides a flat detection plane and has good structural stability during the detection process [87], which is often used to realize biosensing [88]. Due to the removal of part of the cladding, D-type fiber exposes a more evanescent field to the environment [89]. The structure of D-type fiber is more conducive to the excitation of LSPR to realize sensing, so it is also used more in connection with new sensing structures.

**Figure 13:**
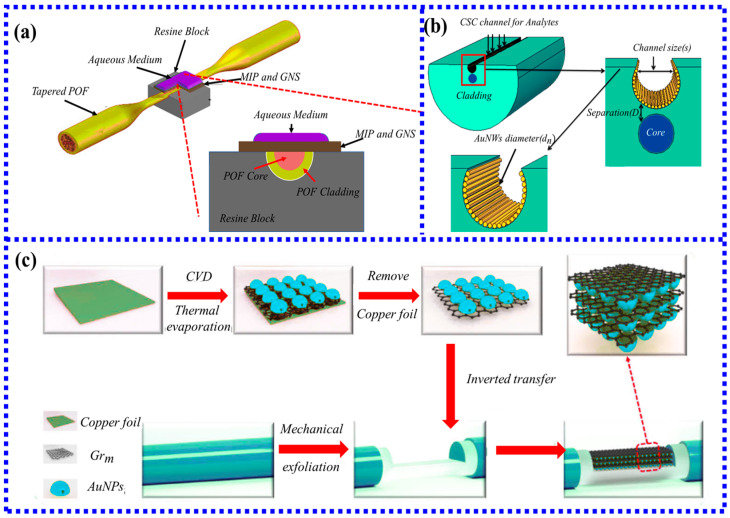
Schematic diagram of the optical sensor system based on LSPR in D-type fiber: (**a**) tapered platform with sensing layer of five-branched gold nanostars and molecularly imprinted polymers [90]. Copyright 2023, Elsevier; (**b**) fiber-optic sensor covered with multiple Au nanowires [91]. (**c**) Schematic of the preparation procedure of n*(Grm/AuNPs)/D-POF [92].

So far, three different methods for sensitizing D-fiber sensors have been reported: combination with D-fiber tapering (Figure 13a), nanowires (Figure 13b), and three-dimensional hybrid structures (Figure 13c). The first sensitization method is the easiest and requires the simplest setup. In 2015, a new method [90] for enhancing sensitivity by combining D-type optical fiber tapered processing with molecularly imprinted polymers was reported to realize the detection of 2,4,6-trinitrotoluene. Using this tapered D-type optical fiber sensor, the sensitivity of the target 2,4,6-trinitrotoluene is 8.3 × 10^5^ nm/M. In addition to using the tapered processing method, the sensor also chooses a combination of special nanoparticles in the shape of a five-pointed star. This type of sensitization method has a major disadvantage. The D-type optical fiber is prepared by destroying the structure of the optical fiber to a certain extent. After the tapering treatment, the sensor intensity will be further reduced. The second method of sensitization is through incorporation with gold nanowires [91]. Compared with gold nanoparticles, gold nanowires are larger and can provide more binding sites. Compared with normal LSPR fiber-optic sensors, the sensitivity can be increased by more than 10 times. However, it is a pity that this method only exists in simulation, and there is no experimental verification. The third mechanism is the use of three-dimensional hybrid structures to achieve sensitization. The mechanism of this approach is to employ the enhancement of resonant coupling through multilayer structures to achieve sensitization. A 3D hybrid structure [92] of multilayer graphene/gold nanoparticle stacks was recently reported using homogeneous plasmonic hybridization to achieve a stronger hot spot effect at the tip and facilitate the coupling of plasmonic excitations. By using the optimal number of stacked layers, the sensor successfully detected the glucose concentration with a sensitivity of 1317.61 nm/RIU. Although the fabrication of the multilayer graphene and gold nanoparticle sensor in this work is very complex, it demonstrates the potential of the 3D hybrid structure as a biomimetic sensor for multiplexing.

Table 2 summarizes the performance, advantages, and disadvantages of specially shaped fiber-optic sensors in recent years. This section summarizes special shape fiber-optic sensors based on LSPR. These special structures show significant advantages in improving the sensitivity and detection of trace reagents. With the development of materials and advanced microfabrication techniques, it is not difficult to foresee the great potential of LSPR fiber-optic sensors with special shapes in label-free optical biosensing. The research on these sensors also lays a solid foundation for practical application.

### 3.3. LSPR Biosensors Based on Specialty Optical Fibers

With the increase of types of specialty fibers and the development of microfabrication technology, LSPR fiber-optic sensors using various specialty fibers and microfabrication technologies have been developed. In this section, we only discuss specially designed or microfabricated fibers, including hollow core fiber (HCF), multi-core fiber (MCF), microstructured optical fiber (MOF), plastic optical fiber (POF), and photosensitive fiber (PSF). LSPR fiber-optic biosensors based on specialty fibers have some advantages that others do not. For example, HCF can coat the interior of the channel with gold nanoparticles for biosensing [45]. In this way, closed measurement without encapsulating can be achieved to reduce the interference of the external environment and simplify experimental steps. These LSPR fiber-optic biosensors using specialty fibers can be well used in the field of biosensing due to their good biocompatibility and other advantages.

#### 3.3.1. LSPR Fiber-Optic Biosensor Based on HCF

**Figure 14:**
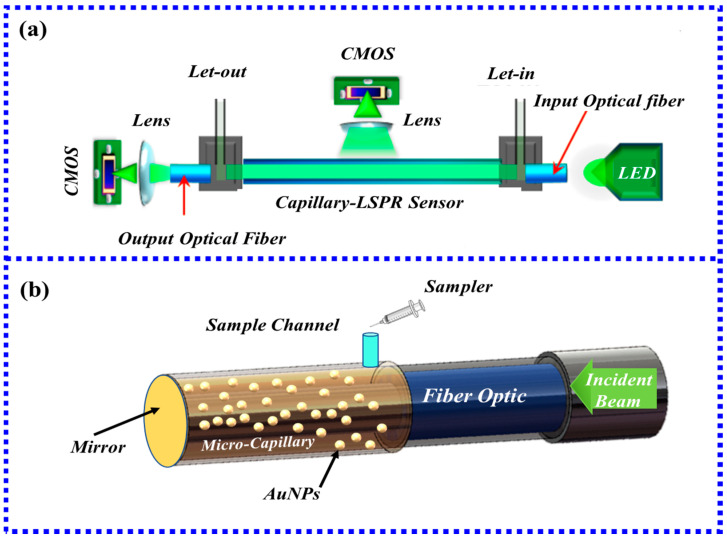
(**a**) Schematic diagram of the plasmonic sensing device based on capillary LSPR sensor [95]; (**b**) Schematic diagram of reflective structure based on HCF.

HCF is a specialty fiber without a solid core structure. The special structure of the HCF determines that the internal air channel can be used as the flow channel of the liquid, thereby reducing the interference of the liquid by the external environment. The light can be transmitted in the tube wall of HCF, thereby generating an evanescent field at the interface between the tube wall and the environment. Compared with the evanescent field generated at the interface between the core and the cladding, the evanescent field of the HCF is more effective because it directly interacts with the metal nanoparticles on the surface. Due to the simple structure, the ability to achieve optofluidic control, and the ease of preparation, LSPR fiber biosensors based on hollow-core fibers have become one of the most important optical biomass detection methods. Besides conventional detectors (e.g., halogen lamps and spectrometers), CMOS-based fiber-optic sensors are also used for LSPR sensing. Due to the flexibility and operability of CMOS-based LSPR fiber-optic biosensors, optimal performance detection, as well as real-time data acquisition, data storage, and data processing, can be achieved. For example, by connecting two CMOS [95] on the tube wall and one end face of the hollow-core fiber, two CMOS image sensors are used to monitor the transmitted light from the output fiber and the scattered light from the side wall of the capillary, respectively, as shown in Figure 14a. When the protein to be tested flows from one end to the other, the concentration of the adsorbed analyte in the solution is determined by the light intensity captured by the CMOS.

In addition to coating the inner side of the hollow fiber with gold nanoparticles to excite LSPR, the outer side of the hollow fiber can also be combined with gold nanoparticles. As shown in Figure 14b, the light from the light source and the light emitted to the spectrometer can pass through the fiber and reflect at the end face of the fiber to excite the gold nanoparticles on the outer surface of the hollow fiber to generate LSPR. Specific detection of human IgG was achieved with a limit of detection (LOD) of 3 nM [96] using this fiber-optic biosensor based on the excitation of LSPR outside of a hollow-core fiber.

HCF-based biosensors have a series of advantages. For example, biosensors based on HCF with microfluidic channels offer a solution for the integration of multicomponent liquids. However, the simple use of HCF as a microfluidic channel requires the use of devices such as image sensors to monitor scattered light. In this way, the scattered light exposed to the environment is easily disturbed by the environment. If a simple observation device is used, the fabrication process of the sensor is extremely complicated, which is a major obstacle to the application of such sensors. Furthermore, the uniform coating of gold nanoparticles onto hollow-core optical fibers should be a major concern for improving sensor performance and stability.

#### 3.3.2. LSPR Fiber-Optic Biosensor Based on MCF

MCF is composed of fiber cladding and multiple fiber cores. This structure has many advantages, such as high sensitivity, compact structure, and low loss. Generally speaking, the core of MCF is smaller than that of ordinary fiber. When light is transmitted from SMF to MCF, multiple modes are excited. The excited mode is extremely sensitive to changes in the surrounding environment of the MCF cladding, resulting in an ultrasensitive sensor [97]. Generally speaking, to increase the strength of the evanescent field, it is necessary to perform hydrofluoric acid etching on the MCF [98] to increase the core area to realize mode coupling or to use the taper processing method to increase the field strength of the evanescent field.

**Figure 15:**
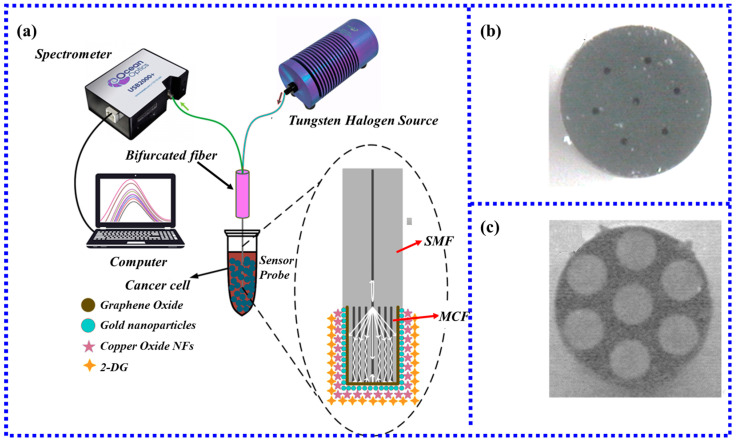
(**a**) Schematic diagram of the MCF-based LSPR cell sensing experimental device [99]; (**b**) MCF cross-section of multicore fiber before HF corrosion [99]; (**c**) MCF cross-section view after HF corrosion [99]. Copyright 2023, Elsevier.

In 2020, Ragini Singh et al. [99] first prepared an LSPR miniature fiber-optic biosensor using MCF to detect cancer cells. Figure 15a shows the MCF-based LSPR cytosensor test setup. Figure 15b shows the cross-section of the original MCF, and Figure 15c shows the cross-sectional view after hydrofluoric acid etching. The structure also employed graphene oxide and copper oxide flowers to increase surface area and biocompatibility. The results show that the MCF-based cytosensors for HepG2, Hepa1 6, A549, MCF-7, LO2, and normal canine fibroblasts have detection limits of 3, 2, 2, 2, 4, and 10 cells/Ml, respectively. Furthermore, the expression of glucose transporter efficiently detected cancer cells and successfully differentiated them from normal cells. Santosh Kumar et al. [94] used the same MCF structure to detect Shigella. The results show that the detection limit is 1.56 CFU/mL. These experimental results demonstrate that MCF-based sensors can be used for detection in multiple biological fields.

In addition to using corrosion to increase sensor performance, it is also possible to improve sensor performance by tapering the MCF. In 2021, Zhu et al. [100] prepared sensing structures of MMF-tapered MCF-MMF and MMF-etched MCF-MMF to realize the specific detection of acetylcholine. The results show that the RI sensitivity and the detection limit of acetylcholine of the tapered MCF are 0.062 nm/uM and 14.28 uM, respectively, which are better than 0.012 nm/uM and 71.3 uM etched by hydrofluoric acid. A new method is proposed to improve the structural properties of MCF.

MCF-based LSPR biosensors usually have the advantages of strong stability, high sensitivity to environmental parameters, and resistance to electromagnetic interference, so they have been widely used in many fields of biology. At the same time, HCF sensors solve the problem of the fragility of micro-nano fibers and also provides a solution for the realization of living cell sensing.

#### 3.3.3. LSPR Fiber-Optic Biosensor Based on MOF

Microstructured fiber (MOF) is also known as photonic crystal fiber (PCF). Different from traditional step-type fibers, MOFs are usually composed of a single medium and a microstructured cladding composed of air holes closely arranged in two-dimensional directions. MOF breaks through the limitations of traditional optical fibers with its unique structure and light transmission method. This kind of fiber provides a new development opportunity for the development of fiber-optic technology and application fields. The air holes of the MOF can be used as microfluidic channels to pass liquids and are, therefore, very suitable for biosensing. In addition, the MOF is made of a single material, so the flexible structural design endows the MOF with superior properties that traditional fibers cannot match, such as no-cut-off single-mode properties, high birefringence properties, refillable properties, and dispersion controllability characteristics [101], etc.

**Figure 16:**
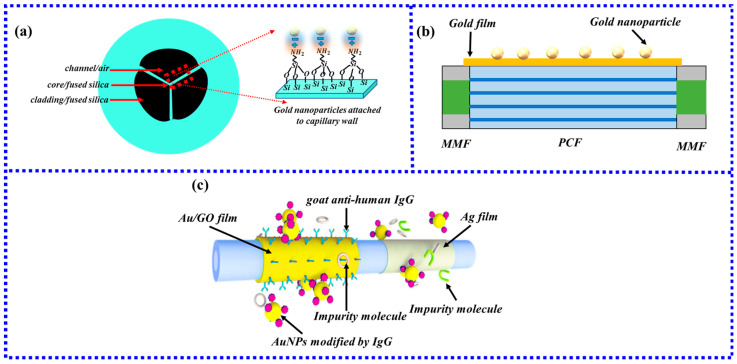
(**a**) Cross-sectional view of the MOF coated with gold nanoparticles on the inner wall. (**b**) Schematic diagram of the single-channel sensor structure; (**c**) Schematic diagram of the dual-channel sensor structure [105]. Copyright 2023, Elsevier.

The use of MOF for sensor design can be mainly divided into two types: surface sensing and internal sensing of MOF. Surface sensing on MOF, as the name suggests, is to realize the measurement of the substance to be tested by functionalizing the surface of the MOF. Internal sensing is mainly realized by coating nanoparticles in the air holes of MOF to excite LSPR to realize sensing. The main advantage of internal sensing is that the position and number of analyte channels can be selected in multiple air holes of MOF to improve the sensing performance [102]. However, there are many types of MOF, and the diameter of air holes ranges from a few microns to hundreds of microns. Andrea Csaki et al. [103] proposed a technology that combined microfluidics and self-assembled monolayer technology to achieve the deposition of nanoparticle layers with a longitudinal uniform particle density of several meters in the interior of the MOF. The RI sensitivity of the fiber-optic sensor is 78 nm/RIU, and this method lays a solid foundation for the sensing of MOF in fiber-optic biology. The radius of the fan-shaped air hole of the MOF used in this structure is about 20 um or more, and the solution can be smoothly passed under pressure to realize the coating of gold nanoparticles. The cross-sectional view of the fiber is shown in Figure 16a. However, it is very difficult to deposit a metal film inside the pores with a diameter of several micrometers of the MOF.

Currently, the commonly used sensing method uses both the SPR principle and LSPR principle to realize the external biomass sensing of MOF. In 2018, Wang et al. [104] proposed a fiber-optic biosensor based on MMF-PCF-MMF for human IgG detection. The schematic diagram of the sensing probe is shown in Figure 16b. Here, the LSPR generated by gold nanoparticles is used to achieve sensitization, and the RI sensitivity is 3915 nm/RIU, which is six times higher than that of the sensor without modified gold nanoparticles. In addition, the sensor modified by the mixture of gold nanoparticles and protein A has a detection limit of 6.3 times lower than that of the sensor without gold nanoparticle modification. This method proves that the LSPR excited by gold nanoparticles plays an important role in improving the sensitivity of the sensor and reducing the detection limit. A dual-sensing channel fiber-optic biosensor [105] was proposed to achieve the specific detection of human IgG while eliminating measurement errors caused by non-specific binding and temperature cross-sensitivity. The sensing channel is shown in Figure 16c. The above results show that the electric field coupling sensor between SPR and LSPR based on MOF fiber has many advantages, such as simple structure, high sensitivity, production feasibility, and extremely sensitive results. This kind of sensor has a wide range of applications in biochemical and biological analyte detection.

The unique structure of the MOF structure makes it have some characteristics that traditional optical fibers do not have. For example, the pore structure of MOFs provides natural channels for material filling. The RI properties of the filling material can directly affect the light-guiding properties of light. By studying the photoconductive properties of the filled MOF, a large amount of information about the interaction between the filled medium and light can be obtained. In terms of biosensing, high-sensitivity detection of biomolecules can be achieved. In addition, because the air holes of the MOF can be selectively filled, multiple materials can be filled in the same MOF fiber to realize multi-parameter detection.

#### 3.3.4. LSPR Fiber-Optic Biosensor Based on POF

As a special optical fiber with good mechanical properties, POF has the characteristics of low manufacturing cost, good toughness, lightweight, easy processing, large diameter, and low splicing loss [106], so it has received extensive attention in this field. Compared with silica fibers that need to be bent at high temperatures, POFs only need to be bent at low temperatures, so POFs are easier to make optical fibers with specific shapes for sensing, which is an important condition for mass production.

**Figure 17:**
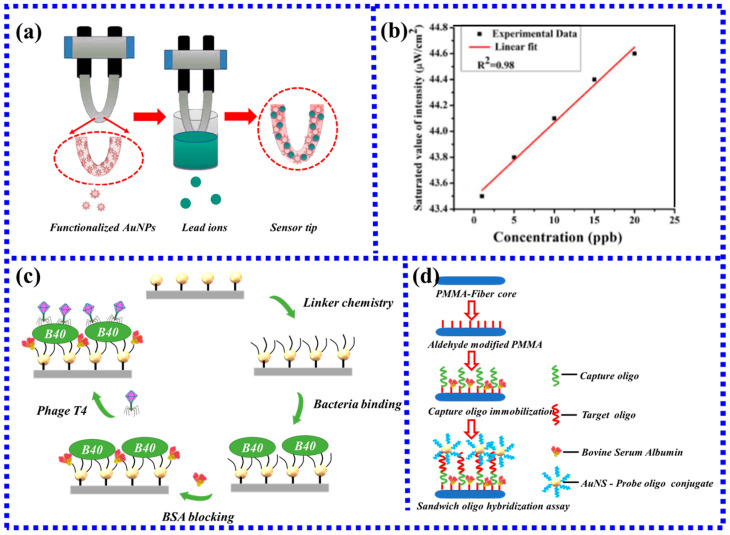
(**a**) The process of the probe capturing Pb^2+^ [107]. (**b**) Linear fit for intensity spectrum [107]. (**c**) Functionalization of *E. coli* captured by T4 phage; (**d**) Assay scheme of oligonucleotides captured by sandwich DNA hybridization.

Bijoy Sankar Boruah [107] first used a U-shaped POF LSPR fiber sensor to detect Pb^2+^ in water. The developed sensor coated with oxalic acid-functionalized AuNPs can selectively bind Pb^2+^ ions, and the interaction mechanism is shown in Figure 17a. The mechanism of Pb^2+^ detection is that the free carboxyl group of oxalic acid can interact with Pb^2+^. Since oxalic acid has two carboxyl groups, one carboxyl group is fixed on the optical fiber through functionalization, and the other carboxyl group is free in the environment to interact with Pb^2+^. When the fiber-optic probe is immersed in the test solution, the intensity does not change on a large scale for about 20 min and then gradually stabilizes within 5 min. Figure 17b shows that the variation of the intensity is linear with the Pb^2+^ concentration, the linear range is determined to be 1–20 ppb, and the limit of detection (LoD) is 2.1 ppb, which is much lower than the WHO guideline value of 10 ppb. The results also prove that the sensor can be well applied in actual production and life. Due to the advantages of high sensitivity and fast response, the U-shaped plastic optical fiber probe has been well used in environmental monitoring, food safety, and other fields.

Besides the detection of pesticide residues and heavy metal ions, POF sensors also achieve rapid detection of most antigens and antibodies. There is an interesting report on a POF biosensor for the detection of *E. coli* using bacteriophage T4 [108]. Although the functionalization steps of the sensor in this work are very complicated, it demonstrates the potential of the POF-based LSPR sensor to develop highly sensitive DNA biosensors, providing a potential label-free biosensor detection method for clinical research. Figure 17c shows the functionalization step of the T4 phage attached to the POF surface to capture *E. coli*. Moreover, Gowri et al. [109] prepared a fiber-optic biosensor using the sandwich DNA hybridization method. As shown in Figure 17d, using this biosensing scheme, the detection limit of 0.2 pM for oligonucleotides was achieved, indicating its potential to develop high-sensitivity DNA biosensors, providing a potential marker-free biosensor detection method for clinical research.

POF-based LSPR biosensors have the advantages of strong stability, fast response, and small size. This kind of sensor simplifies the fabrication steps of the fiber-optic sensing probe to a certain extent, making it better applied to biomedicine.

#### 3.3.5. LSPR Fiber-Optic Biosensor Based on PSF

PSF is a specialty fiber that is sensitive to light. PSFs include highly doped Ge photosensitive fibers, Ge/B co-doped photosensitive fibers, and special doped photosensitive fibers (tantalum-doped photosensitive fibers, cerium-doped photosensitive fiber, tin-doped photosensitive fiber, erbium-doped photosensitive fiber). In this section, we refer to PSF as the Ge-doped photosensitive fiber.

**Figure 18:**
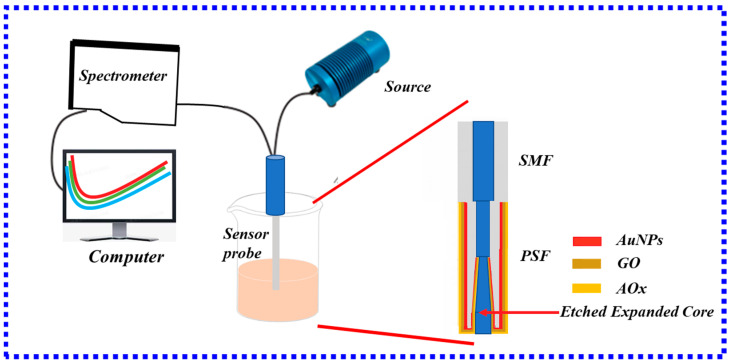
Schematic diagram of the experimental setup for measuring ascorbic acid.

PSF-based biosensors enable rapid detection of most antigens and antibodies. Santosh Kumar et al. [110] proposed a novel high-sensitivity biosensor based on PSF for the detection of ascorbic acid. As shown in Figure 18, the new sensor utilized the biocompatibility of GO to improve its performance. Furthermore, the core of the PSF was expanded in the HF etching, which enhanced the strength of the evanescent field and was more conducive to the detection of ascorbic acid. The experimental results show that the sensor has a wide linear detection range of 1 uM–1 mM, a sensitivity of 3.5%/mM, and a detection limit of 15.12 uM, which is lower than the physiological ascorbic acid level of 40–120 μM in healthy human serum.

Besides ascorbic acid detection, PSF can also effectively detect other biomolecules, which has broad application prospects. In 2022, Wang et al. [111] proposed an MMF-PSF-MMF structure for the high-sensitivity detection of cardiac troponin. The LSPR fiber-optic biosensor was immobilized with graphene oxide, gold nanoparticles, and molybdenum disulfide nanoparticles simultaneously. In the experiment, HF was used to corrode PSF to increase the intensity of the evanescent field, and then graphene oxide, gold nanoparticles, and molybdenum disulfide were sequentially plated on the surface of PSF to effectively collect cardiac troponin. The results show that the biosensor has a linear response of 3.4 pm/(ng/mL) in the concentration range of 0–1000 ng/mL, R2 = 0.928, and the detection limit is 96.2638 ng/mL. The methods provided in this experiment have great benefits in clinical diagnostics, biomedical engineering, and nanotechnology.

Traditional optical fibers have very limited sensitivity to light. Because the RI difference between the core and the cladding is not large, the loss of light is large, which limits the sensitivity. PSF-based biosensors can increase the RI difference between the core and the cladding by increasing the RI of the core, resulting in less optical loss. This makes it shine in the field of biochemistry.

Table 3 summarizes the performance, advantages, and disadvantages of specialty fibers based on the LSPR effect in recent years. In conclusion, LSPR fiber-optic biosensors with different structures have their advantages and disadvantages. These specialty fiber-optic sensors have great potential in optofluidic detection as well as multi-channel detection.

## 4. Outlook

**Figure 19:**
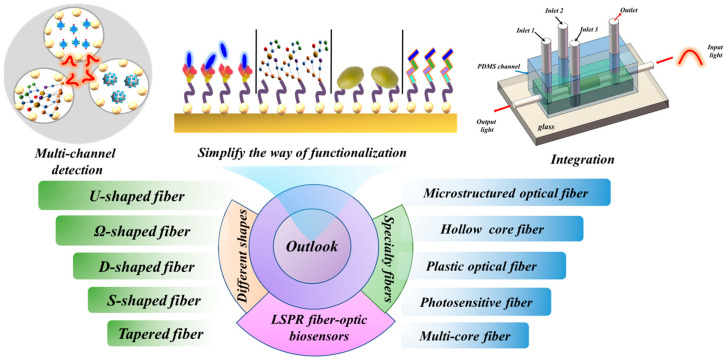
The outlook of LSPR fiber-optic biosensors.

With the high requirements for sensor sensitivity and detection limit, LSPR fiber-optic biosensors have been widely studied and applied. Although progress has been made, there are still some pressing issues to be resolved. Figure 19 shows the outlook of LSPR fiber optic biosensors.

(1) Multi-channel detection. In practical applications, sensors are often interfered with by the environment. Simultaneous detection of multiple channels to eliminate cross-interference is a good solution. In some works, a dual-channel fiber-optic sensor is proposed to eliminate the cross-interference of environmental factors such as temperature [105]. Multi-channel detection using LSPR-based fiber-optic sensors has great potential for high-sensitivity multi-parameter detection. In addition, realizing the detection of multiple substances in multiple microfluidic channels of the same MOF is an important improvement direction for realizing multi-channel detection.

(2) Integration. At present, LSPR fiber-optic biosensors are generally used to measure only one or two substances, and few LSPR fiber-optic biosensors are integrated on a chip to achieve the measurement of multiple biomass. It is worth mentioning that Professor Cusano’s team has made great contributions in the lab on a chip based on LSPR fiber-optic sensors. They made a detailed analysis and summary of this aspect [113,114]. This shows that the application of LSPR-based fiber-optic sensors in the lab on a chip makes LSPR-based LSPR biosensors very suitable for multiplex detection. Besides, Hyeong-Min Kim et al. [49] proposed a sensor that can flow multiple channels into the same sensing area. This setup has great potential for multiple biomass detection. In the future, through the development and use of advanced manufacturing technology, the precise control of LSPR can be realized, and various parameters of the probe can be freely modified to realize integrated detection.

(3) Simplify the way of functionalization. Liu et al. [115] used the flash of the smartphone as a light source and a complementary metal-oxide-semiconductor camera as a detector to monitor the transmission intensity changes at the end face of a fiber-optic sensor. This setup achieves RI measurement in real time. At present, although the detection limit, detection accuracy, and sensitivity of LSPR fiber-optic biosensors have greatly improved in biosensing, the functionalization steps of current fiber-optic biosensors are complicated, and most of the experimental reagents have biological toxicity. So, the present functionalization way can be only operated in the laboratory and cannot be used in real life, and the current limitations are relatively large. In the future, with the continuous development of materials science and nanotechnology, there will be more reagents with simple operations and no toxic effects. These new reagents can be functionalized and routinely measured.

## 5. Conclusions

This paper reviews the research progress of LSPR fiber-optic biosensors. Based on the classification of sensor structures, various biosensors with different structures are reviewed in detail. The main ones include ordinary optical fibers, special shapes, and specialty fibers. The special shapes include tapered, U-type, Ω-type, S-type, and D-type. Specialty fibers include HCF, MCF, MOF, POF, and PSF. Moreover, the advantages, disadvantages, and performance of each type of sensor are analyzed and summarized. Finally, by summarizing the existing deficiencies and future needs of LSPR fiber-optic biosensors, its future development trends are predicted. At present, this research field is growing rapidly. With the further development of fiber-optic sensing technology and functionalized technology, improving the sensitivity and detection limit of sensors has become the main goal of this field. In the near future, LSPR fiber-optic biosensors will go out of the laboratory and be applied in the fields of biomedicine, environmental monitoring, food safety detection, and clinical analysis.

## Figures and Tables

**Table 1:** The recognition method of the target detection substance.

Method	Advantage		Disadvantage	Ref
specific recognition	Antigen-antibody binding	High specificity sensitivity, good stability, low interference from the natural environment, etc.	Combining with the fiber surface complicated	[25]
	Aptamer	Strong selectivity, high affinity	Aptamers target only one target	[26]
	Molecular imprinting	Stable against harsh environments, materials used can be reused, predeterminability, high recognition, and practicability	Templates are not readily available and expensive	[27]
Microbial detection method	Rapid sensitivity test		Narrow detection range	[28]
Physical adsorption of Mesoporous nanospheres	High repeatability and stability		The complicated preparation process, no selectivity	[29]
Polyelectrolyte method	Easy to operate, No complex functionalization process		Difficult requirements on the charge of the measured substance, no selectivity	[30]

**Table 2:** The recognition method of the target detection substance of different shapes sensor.

Shape	Target	Sensor Performance	Advantage	Disadvantage	Ref
Tapered	Glucose	One tapered, sensitivity: 1.06 nm/mM	Ultrahigh sensitivity, superfine diameter.	Fragile structure, Complex optical fiber manufacturing method	[69]
	Alanine aminotransferase	Taper-in-taper, Sensitivity: 4.1 pm/(U/L)			[74]
	Ascorbic acid Ascorbic acid Ascorbic acid	Four tapered, Sensitivity: 1.1 nm/mM Five tapered, Sensitivity: 8.3 nm/mM Eight tapered, Sensitivity: 0.5 nm/mM			[75] [75] [75]
U-type	Cancer cell detection	LOD: 30 cells/mL	Small size, stable structure, Simple preparation process	Only transmissive structure	[93]
Ω-type	MCF-7 cancer cells	LOD: 12 cells/ml	Small size, 2.5 times higher sensitivity than U-type	Only transmissive structure	[80]
	Salmonella typhimurium	LOD: 7.4 CFU/mL			[82]
	MCF-7 cancer cells	LOD: 2.6 cells/ml			[83]
S-type	2,4,6- Trinitrotoluene; 2,4-Dinitrotoluene;	LOD: 10 parts per billion (ppb)	1.5 times higher sensitivity than U-type	Fragile structure	[85]
D-type	Goat human IgG 2,4,6-trinitrotoluene Water-glycerin solutions	LOD: 0.6 μg/mL $\mathrm{Sensitivity}:\;8.3\;\times \;10^{5}$ nm/M RI sensitivity: 84 nm/RIU	Flexible structure and larger sensing platform	Grinding and polishing surface rough	[88] [90]
					[94]

**Table 3:** The recognition method of the target detection substance of different specialty fiber optic sensor.

Type	Target	Sensor Performance	Advantage	Disadvantage	Ref
HCF	Cholesterol	LOD: 25.5 nM	Providing liquid flow channels, saving solution	Complex detection process	[112]
	Human IgG	LOD: 3 nM			[96]
	Transferrin Immunoglobulin G	Range: 0.01–0.15 mg/L Range: 0.01–0.15 mg/L			[95] [95]
MCF	Shigella bacterial HepG2, Hepa1 6, A549, MCF-7, LO2, NCF cell Acetylcholine Creatinine	LOD: 1.56 CFU/mL LOD: 3, 2, 2, 2, 4, 10 cells/mL Sensitivity: 0.062 nm/uM LOD: 14.28 uM Sensitivity: 0.0025 nm/μM LOD: 128.4 μM	High sensitivity to small RI changes, low connection loss, simultaneous measurements on each core	Complex introducing and detecting light from the individual cores	[98]
					[99] [100] [97]
MOF	Refractive Index Solution	RI sensitivity:78 nm/RIU	Providing liquid flow channels, saving solution, temperature not cross interference	The complex optical fiber manufacturing method	[103]
POF	Pb^2+^ DNA *E. coli*	Sensitivity: 0.19 nm/μM LOD: 1 pg/mL Qualitative detection	Low manufacturing cost, good toughness, lightweight, easy processing,	High attenuation, poor heat resistance;	[107] [109] [108]
PSF	Ascorbic acid Cardiac Troponin I	LOD: 15.12 μM Sensitivity: 3.4 pm/(ng/mL), LOD: 96.2638 ng/mL	High sensitivity and low loss	The complex optical fiber manufacturing method	[110]
					[111]

## Data Availability

No new data were created or analyzed in this study. Data sharing is not applicable to this article.

## References

[1] Chang T.-C., Sun A.Y., Huang Y.-C., Wang C.-H., Wang S.-C., Chau L.-K. (2022). Integration of Power-Free and Self-Contained Microfluidic Chip with Fiber Optic Particle Plasmon Resonance Aptasensor for Rapid Detection of SARS-CoV-2 Nucleocapsid Protein. Biosensors.

[2] Li X., Chen N., Zhou X., Zhang Y., Zhao Y., Nguyen L.V., Ebendorff-Heidepriem H., Warren-Smith S.C.J.S., Chemical A.B. (2022). In-situ DNA detection with an interferometric-type optical sensor based on tapered exposed core microstructured optical fiber. Sens. Actuators B Chem..

[3] Sypabekova M., Aitkulov A., Blanc W., Tosi D. (2020). Reflector-less nanoparticles doped optical fiber biosensor for the detection of Case thrombin. Biosens. Bioelectron..

[4] Arjmand M., Aray A., Saghafifar H., Alijanianzadeh M. (2020). Quantitative Analysis of Methyl-Parathion Pesticide in Presence of Enzyme Substrate Using Tapered Fiber Optic Biosensor. IEEE Sens. J..

[5] Mahani M., Alimohamadi F., Torkzadeh-Mahani M., Hassani Z., Khakbaz F., Divsar F., Yoosefian M. (2021). LSPR biosensing for the early-stage prostate cancer detection using hydrogen bonds between PSA and antibody: Molecular dynamic and experimental study. J. Mol. Liq..

[6] Fang S., Song D., Zhu A., Long F. (2020). Nanoporous layer fiber biosensing platform for real time culture- and separation-free detecting bacterial pathogens and measuring their susceptibility to antibiotics. Sens. Actuators B-Chem..

[7] Han S., Zhou X., Tang Y., He M., Zhang X., Shi H., Xiang Y. (2016). Practical, highly sensitive, and regenerable evanescent-wave biosensor for detection of Hg^2+^ and Pb^2+^ in water. Biosens. Bioelectron..

[8] Sharma P., Semwal V., Gupta B.D. (2019). A highly selective LSPR biosensor for the detection of taurine realized on optical fiber substrate and gold nanoparticles. Opt. Fiber Technol..

[9] Wang B.-T., Wang Q. (2018). An interferometric optical fiber biosensor with high sensitivity for IgG/anti-IgG immunosensing. Opt. Commun..

[10] Li X., Li F., Zhou X., Zhang Y., Nguyen L.V., Warren-Smith S.C., Zhao Y. (2022). Measurement. Optical fiber DNA biosensor with temperature monitoring based on double microcavities Fabry-Perot interference and Vernier combined effect. IEEE Trans. Instrum. Meas..

[11] Guo Z., Qin Y., Chen P., Hu J., Zhou Y., Zhao X., Liu Z., Fei Y., Jiang X., Wu X. (2020). Hyperboloid-Drum Microdisk Laser Biosensors for Ultrasensitive Detection of Human IgG. Small.

[12] Wang Q., Jing J.-Y., Wang B.-T. (2019). Highly Sensitive SPR Biosensor Based on Graphene Oxide and Staphylococcal Protein A Co-Modified TFBG for Human IgG Detection. IEEE Trans. Instrum. Meas..

[13] Li X., Gong P., Zhao Q., Zhou X., Zhang Y., Zhao Y.J.S., Chemical A.B. (2022). Plug-in optical fiber SPR biosensor for lung cancer gene detection with temperature and pH compensation. Sens. Actuators B Chem..

[14] Semwal V., Gupta B.D. Localized Surface Plasmon Resonance Based Tapered Fiber Optic Ethanol Sensor. Proceedings of the Conference on Lasers and Electro-Optics Europe/European Quantum Electronics Conference (CLEO/Europe-EQEC).

[15] You Z., Qiu Q., Chen H., Feng Y., Wang X., Wang Y., Ying Y. (2020). Laser-induced noble metal nanoparticle-graphene composites enabled flexible biosensor for pathogen detection. Biosens. Bioelectron..

[16] Agnarsson B., Ingthorsson S., Gudjonsson T., Leosson K. (2009). Evanescent-wave fluorescence microscopy using symmetric planar waveguides. Opt. Express.

[17] Zhang X., Zhang J., Zhang F., Yu S. (2017). Probing the binding affinity of plasma proteins adsorbed on Au nanoparticles. Nanoscale.

[18] Velusamy P., Kumar G.V., Jeyanthi V., Das J., Pachaiappan R. (2016). Bio-Inspired Green Nanoparticles: Synthesis, Mechanism, and Antibacterial Application. Toxicol. Res..

[19] Luo B., Xu Y., Wu S., Zhao M., Jiang P., Shi S., Zhang Z., Wang Y., Wang L., Liu Y. (2018). A novel immunosensor based on excessively tilted fiber grating coated with gold nanospheres improves the detection limit of Newcastle disease virus. Biosens. Bioelectron..

[20] Paul D., Dutta S., Saha D., Biswas R. (2017). LSPR based Ultra-sensitive low cost U-bent optical fiber for volatile liquid sensing. Sens. Actuators B-Chem..

[21] Xu Y., Xiong M., Yan H. (2021). A portable optical fiber biosensor for the detection of zearalenone based on the localized surface plasmon resonance. Sens. Actuators B-Chem..

[22] Zhou X., Gong P., Ai Y., Hou Y., Li X. (2023). Miniature optical fiber DNA hybridization sensor with temperature compensation using a gold nanofilm-coated optical fiber. IEEE Sens. J..

[23] Chen S., Zhang C., Wang J., Li N., Song Y., Wu H., Liu Y. (2023). A Fiber Bragg Grating Sensor Based on Cladding Mode Resonance for Label-Free Biosensing. Biosensors.

[24] Abakah B. (2023). Non-Destructive Imaging of Phytosulfokine Trafficking in Plants Using Fiber-Optic Fluorescence Microscopy. Master’s Thesis.

[25] Liu Z., Zhang W., Zhang X., Wang S., Xia Z., Guo X., Zhao Y., Wang P., Wang X.-H. (2023). Microstructured Optical Fiber-Enhanced Light–Matter Interaction Enables Highly Sensitive Exosome-Based Liquid Biopsy of Breast Cancer. Anal. Chem..

[26] Deng C., Ranathunga C., Banerjee P.P., Chen X., Zhong J.F., Sinha U.K. (2022). Practical tapered optical fiber system for in-situ label-free sensing of various antigens. Opt. Eng..

[27] Zhao S., Huang J., Li D., Yang L. (2022). Aptamer-based chemiluminescent optical fiber immunosensor with enhanced signal amplification for ultrasensitive detection of tumor biomarkers. Biosens. Bioelectron..

[28] Nag P., Sadani K., Mukherji S., Mukherji S. (2020). Beta-lactam antibiotics induced bacteriolysis on LSPR sensors for assessment of antimicrobial resistance and quantification of antibiotics. Sens. Actuators B-Chem..

[29] Ding M., Huang Y., Guo T., Sun L.-P., Guan B.-O. (2016). Mesoporous nanospheres functionalized optical microfiber biosensor for low concentration neurotransmitter detection. Opt. Express.

[30] Yuan H., Ji W., Chu S., Liu Q., Guang J., Sun G., Zhang Y., Han X., Masson J.-F., Peng W. (2020). Au nanoparticles as label-free competitive reporters for sensitivity enhanced fiber-optic SPR heparin sensor. Biosens. Bioelectron..

[31] Sung H.K., Oh S.Y., Park C., Kim Y. (2013). Colorimetric Detection of Co^2+^ Ion Using Silver Nanoparticles with Spherical, Plate, and Rod Shapes. Langmuir.

[32] Zhao S., Cheng Z., Wang S., Hao H., Fang Y. (2021). Maintaining the localized surface plasmon resonance of copper nanoparticles by defective TiO2 thin films. Appl. Phys. A-Mater. Sci. Process..

[33] Liu R., Jiang L., Yu Z., Jing X., Liang X., Wang D., Yang B., Lu C., Zhou W., Jin S.J.S. (2021). MXene (Ti3C2Tx)-Ag nanocomplex as efficient and quantitative SERS biosensor platform by in-situ PDDA electrostatic self-assembly synthesis strategy. Sens. Actuators B Chem..

[34] Shao Y., Xu S., Zheng X., Wang Y., Xu W. (2010). Optical Fiber LSPR Biosensor Prepared by Gold Nanoparticle Assembly on Polyelectrolyte Multilayer. Sensors.

[35] Zhang Q., Xue C., Yuan Y., Lee J., Sun D., Xiong J. (2012). Fiber Surface Modification Technology for Fiber-Optic Localized Surface Plasmon Resonance Biosensors. Sensors.

[36] Khatri A., Punjabi N., Ghosh D., Maji S., Mukherji S. Detection of α-Synuclein, Marker for Parkinson’s disease using Localized Surface Plasmon Resonance Fiber Optic Sensor. Proceedings of the International Conference on Fibre Optics and Photonics.

[37] Tseng Y.-T., Li W.-Y., Yu Y.-W., Chiang C.-Y., Liu S.-Q., Chau L.-K., Lai N.-S., Chou C.-C. (2020). Fiber Optic Particle Plasmon Resonance Biosensor for Label-Free Detection of Nucleic Acids and Its Application to HLA-B27 mRNA Detection in Patients with Ankylosing Spondylitis. Sensors.

[38] Vu Thi H., Nguyen Tran Truc P., Nguyen Tien T., Nguyen Thuy A., Vu Dinh L., Do Hung M., Tran Thi Kim C., Ngoc Xuan Dat M., Viet-Duc P., Nhu Hoa Thi T. (2021). Gold Nanoparticles Modified a Multimode Clad-Free Fiber for Ultrasensitive Detection of Bovine Serum Albumin. J. Nanomater..

[39] Dhara P., Kumar R., Binetti L., Nguyen H.T., Alwis L.S., Sun T., Grattan K.T.V. (2019). Optical Fiber-Based Heavy Metal Detection Using the Localized Surface Plasmon Resonance Technique. IEEE Sens. J..

[40] Yang Q., Zhang X., Kumar S., Singh R., Zhang B., Bal C., Pu X. (2020). Development of Glucose Sensor Using Gold Nanoparticles and Glucose-Oxidase Functionalized Tapered Fiber Structure. Plasmonics.

[41] Kumar S., Kaushik B.K., Singh R., Chen N.-K., Yang Q.S., Zhang X., Wang W., Zhang B. (2019). LSPR-based cholesterol biosensor using a tapered optical fiber structure. Biomed. Opt. Express.

[42] Hu B., Kong F., Gao X., Jiang L., Li X., Gao W., Xu K., Tang B. (2018). Avoiding Thiol Compound Interference: A Nanoplatform Based on High-Fidelity Au-Se Bonds for Biological Applications. Angew. Chem.-Int. Ed..

[43] Piwonski I., Grobelny J., Cichomski M., Celichowski G., Rogowski J. (2005). Investigation of 3-mercaptopropyltrimethoxysilane self-assembled monolayers on Au(111) surface. Appl. Surf. Sci..

[44] Jia S., Bian C., Sun J., Xia S. Gold nanospheres-coated lspr fiber sensor with high ri sensitivity by a rapid fabricating method. Proceedings of the 2018 IEEE 13th Annual International Conference on Nano/Micro Engineered and Molecular Systems (NEMS).

[45] Goicoechea J., Rivero P.J., Sada S., Arregui F.J. (2019). Self-Referenced Optical Fiber Sensor for Hydrogen Peroxide Detection Based on LSPR of Metallic Nanoparticles in Layer-by-Layer Films. Sensors.

[46] Lu M., Peng W., Lin M., Wang F., Zhang Y. (2021). Gold Nanoparticle-Enhanced Detection of DNA Hybridization by a Block Copolymer-Templating Fiber-Optic Localized Surface Plasmon Resonance Biosensor. Nanomaterials.

[47] Kim H.-M., Jeong D.H., Lee H.-Y., Park J.-H., Lee S.-K. (2019). Improved stability of gold nanoparticles on the optical fiber and their application to refractive index sensor based on localized surface plasmon resonance. Opt. Laser Technol..

[48] Lu M., Zhu H., Lin M., Wang F., Hong L., Masson J.-F., Peng W.J.S., Chemical A.B. (2021). Comparative study of block copolymer-templated localized surface plasmon resonance optical fiber biosensors: CTAB or citrate-stabilized gold nanorods. Sens. Actuators B Chem..

[49] Kim H.-M., Park J.-H., Jeong D.H., Lee H.-Y., Lee S.-K. (2018). Real-time detection of prostate-specific antigens using a highly reliable fiber-optic localized surface plasmon resonance sensor combined with micro fluidic channel. Sens. Actuators B-Chem..

[50] Kim H.-M., Uh M., Jeong D.H., Lee H.-Y., Park J.-H., Lee S.-K. (2019). Localized surface plasmon resonance biosensor using nanopatterned gold particles on the surface of an optical fiber. Sens. Actuators B-Chem..

[51] Dhawan A., Gerhold M., Madison A., Fowlkes J., Russell P.E., Vo-Dinh T., Leonard D.N. (2009). Fabrication of Nanodot Plasmonic Waveguide Structures Using FIB Milling and Electron Beam-Induced Deposition. Scanning.

[52] Hong Y., Zhao D., Wang J., Lu J., Yao G., Liu D., Luo H., Li Q., Qiu M. (2020). Solvent-free nanofabrication based on ice-assisted electron-beam lithography. Nano Lett..

[53] Kim H.-M., Nam K.-T., Lee S.-K., Park J.-H. (2018). Fabrication and measurement of microtip-array-based LSPR sensor using bundle fiber. Sens. Actuators A-Phys..

[54] Bresin M., Nehru N., Hastings J.T. Focused electron-beam induced deposition of plasmonic nanostructures from aqueous solutions. Proceedings of the Advanced Fabrication Technologies for Micro/Nano Optics and Photonics VI.

[55] Dhawan A., Gerhold M., Vo-Dinh T.J.N. (2007). Theoretical simulation and focused ion beam fabrication of gold nanostructures for surface-enhanced Raman scattering (SERS). Nanobiotechnology.

[56] Hoeflich K., Jurczyk J., Zhang Y., Puydinger dos Santos M.V., Goetz M., Guerra-Nunez C., Best J.P., Kapusta C., Utke I. (2017). Direct Electron Beam Writing of Silver-Based Nanostructures. ACS Appl. Mater. Interfaces.

[57] Wang S., Sun Y.M., Wang Q., White J.M. (2004). Electron-beam induced initial growth of platinum films using Pt(PF3)(4). J. Vac. Sci. Technol. B.

[58] Chien M.-H., Shawrav M.M., Hingerl K., Taus P., Schinnerl M., Wanzenboeck H.D., Schmid S. (2021). Analysis of carbon content in direct-write plasmonic Au structures by nanomechanical scanning absorption microscopy. J. Appl. Phys..

[59] Flatae A.M., Tantussi F., Messina G.C., Mohammadi A., De Angelis F., Agio M. (2017). Plasmonic Gold Nanocones in the Near-Infrared for Quantum Nano-Optics. Adv. Opt. Mater..

[60] Vaiano P., Quero G., Fienga F., Di Meo V., Casolaro P., Campajola L., Breglio G., Crescitelli A., Esposito E., Cutolo A.J.O. (2023). Characterization of Lab-on-Fiber-based dosimeters in ultra-high dose radiation fields. Opt. Laser Technol..

[61] Keiser G., Xiong F., Cui Y., Shum P.P. (2014). Review of diverse optical fibers used in biomedical research and clinical practice. J. Biomed. Opt..

[62] Khanikar T., De M., Singh V.K.J.P. (2021). A review on infiltrated or liquid core fiber optic SPR sensors. Photonics Nanostruct. Fundam. Appl..

[63] Agrawal N., Saha C., Kumar C., Singh R., Zhang B., Kumar S. (2020). Development of Uric Acid Sensor Using Copper Oxide and Silver Nanoparticles Immobilized SMSMS Fiber Structure-Based Probe. IEEE Trans. Instrum. Meas..

[64] Sanders M., Lin Y., Wei J., Bono T., Lindquist R.G. (2014). An enhanced LSPR fiber-optic nanoprobe for ultrasensitive detection of protein biomarkers. Biosens. Bioelectron..

[65] Malara P., Crescitelli A., Di Meo V., Giorgini A., Avino S., Esposito E., Ricciardi A., Cusano A., Rendina I., De Natale P.J.S. (2018). Resonant enhancement of plasmonic nanostructured fiber optic sensors. Sens. Actuators B Chem..

[66] Kim H.-M., Park J.-H., Lee S.-K. (2019). Fiber optic sensor based on ZnO nanowires decorated by Au nanoparticles for improved plasmonic biosensor. Sci. Rep..

[67] Kim H.-M., Park J.-H., Lee S.-K. (2021). Fabrication and measurement of fiber optic localized surface plasmon resonance sensor based on gold nanoparticle dimer. Spectrochim. Acta Part A-Mol. Biomol. Spectrosc..

[68] Flauraud V., Regmi R., Winkler P.M., Alexander D.T.L., Rigneault H., van Hulst N.F., Garcia-Parajo M.F., Wenger J., Brugger J. (2017). In-Plane Plasmonic Antenna Arrays with Surface Nanogaps for Giant Fluorescence Enhancement. Nano Lett..

[69] Yang Q., Zhu G., Singh L., Wang Y., Singh R., Zhang B., Zhang X., Kumar S. (2020). Highly sensitive and selective sensor probe using glucose oxidase/gold nanoparticles/graphene oxide functionalized tapered optical fiber structure for detection of glucose. Optik.

[70] Dalila N.R., Arshad M.K.M., Gopinath S.C.B., Norhaimi W.M.W., Fathil M.F.M. (2019). Current and future envision on developing biosensors aided by 2D molybdenum disulfide (MoS2) productions. Biosens. Bioelectron..

[71] Esfahani Monfared Y. (2020). Overview of Recent Advances in the Design of Plasmonic Fiber-Optic Biosensors. Biosensors.

[72] Semwal V., Gupta B.D. (2019). Experimental studies on the sensitivity of the propagating and localized surface plasmon resonance-based tapered fiber optic refractive index sensors. Appl. Opt..

[73] Wang J., Luo Z., Zhou M., Ye C., Fu H., Cai Z., Cheng H., Xu H., Qi W. (2012). Evanescent-Light Deposition of Graphene Onto Tapered Fibers for Passive Q-Switch and Mode-Locker. IEEE Photonics J..

[74] Wang Z., Singh R., Marques C., Jha R., Zhang B., Kumar S. (2021). Taper-in-taper fiber structure-based LSPR sensor for alanine aminotransferase detection. Opt. Express.

[75] Zhu G., Agrawal N., Singh R., Kumar S., Zhang B., Saha C., Kumar C. (2020). A novel periodically tapered structure-based gold nanoparticles and graphene oxide—Immobilized optical fiber sensor to detect ascorbic acid. Opt. Laser Technol..

[76] Wen H.-Y., Hsu H.-C., Tsai Y.-T., Feng W.-K., Lin C.-L., Chiang C.-C.J.P. (2021). U-shaped optical fiber probes coated with electrically doped GQDs for humidity measurements. Polymers.

[77] Sadani K., Nag P., Mukherji S. (2019). LSPR based optical fiber sensor with chitosan capped gold nanoparticles on BSA for trace detection of Hg (II) in water, soil and food samples. Biosens. Bioelectron..

[78] Halkare P., Punjabi N., Wangchuk J., Nair A., Kondabagil K., Mukherji S. (2019). Bacteria functionalized gold nanoparticle matrix based fiber-optic sensor for monitoring heavy metal pollution in water. Sens. Actuators B-Chem..

[79] Arcas A.d.S., Dutra F.d.S., Allil R.C.S.B., Werneck M.M. (2018). Surface Plasmon Resonance and Bending Loss-Based U-Shaped Plastic Optical Fiber Biosensors. Sensors.

[80] Luo Z., Xu Y., He L., He F., Wu J., Huang Z., Tian Y., Li Y., Duan Y. (2021). Development of a rapid and ultra-sensitive cytosensor: Ω-shaped fiber optic LSPR integrated with suitable AuNPs coverage. Sens. Actuators B Chem..

[81] Xu Y., Luo Z., Chen J., Huang Z., Wang X., An H., Duan Y. (2018). Omega-Shaped Fiber-Optic Probe-Based Localized Surface Plasmon Resonance Biosensor for Real-Time Detection of Salmonella Typhimurium. Anal. Chem..

[82] Li Y., Wang X., Ning W., Yang E., Li Y., Luo Z., Duan Y. (2022). Sandwich method-based sensitivity enhancement of Ω-shaped fiber optic LSPR for time-flexible bacterial detection. Biosens. Bioelectron..

[83] He L., He F., Feng Y., Wang X., Li Y., Tian Y., Gao A., Zhang P., Qi X., Luo Z. (2021). Hybridized nanolayer modified Omega-shaped fiber-optic synergistically enhances localized surface plasma resonance for ultrasensitive cytosensor and efficient photothermal therapy. Biosens. Bioelectron..

[84] Chauhan S.K., Tharion J., Punjabi N., Sharma D.K., Mukherji S. A comparison of S-shaped and U-shaped optical fiber sensors. Proceedings of the Optical Sensors.

[85] Chauhan S., Punjabi N., Sharma D., Mukherji S. (2016). Evanescent wave absorption based S-shaped fiber-optic biosensor for immunosensing applications. Procedia Eng..

[86] Dimpi P., Biswas R. (2022). Clad modified varying geometries of fiber optic LSPR sensors towards detection of hazardous volatile liquids and their comparative analysis. Environ. Technol. Innov..

[87] Gong W., Jiang S., Li Z., Li C., Xu J., Pan J., Huo Y., Man B., Liu A., Zhang C. (2019). Experimental and theoretical investigation for surface plasmon resonance biosensor based on graphene/Au film/D-POF. Opt. Express.

[88] Virk J.K., Das S., Kaler R.S., Singh H., Kundu T. (2022). D-shape optical fiber probe dimension optimization for LSPR based bio-sensor. Opt. Fiber Technol..

[89] Liu G., Feng D. (2016). Evanescent wave analysis and experimental realization of refractive index sensor based on D-shaped plastic optical fiber. Optik.

[90] Cennamo N., Dona A., Pallavicini P., D’Agostino G., Dacarro G., Zeni L., Pesavento M. (2015). Sensitive detection of 2,4,6-trinitrotoluene by tridimensional monitoring of molecularly imprinted polymer with optical fiber and five-branched gold nanostars. Sens. Actuators B-Chem..

[91] Pathak A.K., Rahman B.M.A., Singh V.K., Kumari S. (2019). Sensitivity Enhancement of a Concave Shaped Optical Fiber Refractive Index Sensor Covered with Multiple Au Nanowires. Sensors.

[92] Feng J., Gao J., Yang W., Liu R., Shafi M., Zha Z., Liu C., Xu S., Ning T., Jiang S. (2022). LSPR optical fiber sensor based on 3D gold nanoparticles with monolayer graphene as a spacer. Opt. Express.

[93] Luo Z., Wang Y., Xu Y., Wang X., Huang Z., Chen J., Li Y., Duan Y. (2019). Ultrasensitive U-shaped fiber optic LSPR cytosensing for label-free and in situ evaluation of cell surface N-glycan expression. Sens. Actuators B-Chem..

[94] Cennamo N., D’Agostino G., Donà A., Dacarro G., Pallavicini P., Pesavento M., Zeni L.J.S. (2013). Localized surface plasmon resonance with five-branched gold nanostars in a plastic optical fiber for bio-chemical sensor implementation. Sensors.

[95] Liu Y., Zhang N., Li P., Yu L., Chen S., Zhang Y., Jing Z., Peng W.J.N. (2019). Low-cost localized surface plasmon resonance biosensing platform with a response enhancement for protein detection. Nanomaterials.

[96] Chen S., Liu Y., Yu Q., Peng W. (2020). Microcapillary-based integrated LSPR device for refractive index detection and biosensing. J. Light. Technol..

[97] Li M., Singh R., Marques C., Zhang B., Kumar S. (2021). 2D material assisted SMF-MCF-MMF-SMF based LSPR sensor for creatinine detection. Opt. Express.

[98] Kumar S., Guo Z., Singh R., Wang Q., Zhang B., Cheng S., Liu F.-Z., Marques C., Kaushik B.K., Jha R.J. (2021). MoS_2_ functionalized multicore fiber probes for selective detection of shigella bacteria based on localized plasmon. J. Light. Technol..

[99] Singh R., Kumar S., Liu F.-Z., Shuang C., Zhang B., Jha R., Kaushik B.K. (2020). Etched multicore fiber sensor using copper oxide and gold nanoparticles decorated graphene oxide structure for cancer cells detection. Biosens. Bioelectron..

[100] Zhu G., Wang Y., Wang Z., Singh R., Marques C., Wu Q., Kaushik B.K., Jha R., Zhang B., Kumar S. (2021). Localized Plasmon-Based Multicore Fiber Biosensor for Acetylcholine Detection. IEEE Trans. Instrum. Meas..

[101] Li X., Chen N., Zhou X., Gong P., Wang S., Zhang Y., Zhao Y. (2021). A review of specialty fiber biosensors based on interferometer configuration. J. Biophotonics.

[102] Mollah M.A., Islam M.S. (2021). Novel Single Hole Exposed-Suspended Core Localized Surface Plasmon Resonance Sensor. IEEE Sens. J..

[103] Csaki A., Jahn F., Latka I., Henkel T., Malsch D., Schneider T., Schroeder K., Schuster K., Schwuchow A., Spittel R. (2010). Nanoparticle Layer Deposition for Plasmonic Tuning of Microstructured Optical Fibers. Small.

[104] Wang B.-T., Wang Q. (2018). Sensitivity-Enhanced Optical Fiber Biosensor Based on Coupling Effect Between SPR and LSPR. IEEE Sens. J..

[105] Wang Q., Wang X.-Z., Song H., Zhao W.-M., Jing J.-Y. (2020). A dual channel self-compensation optical fiber biosensor based on coupling of surface plasmon polariton. Opt. Laser Technol..

[106] Kuriki K., Koike Y., Okamoto Y. (2002). Plastic optical fiber lasers and amplifiers containing lanthanide complexes. Chem. Rev..

[107] Boruah B.S., Biswas R. (2018). Localized surface plasmon resonance based U-shaped optical fiber probe for the detection of Pb^2+^ in aqueous medium. Sens. Actuators B-Chem..

[108] Halkare P., Punjabi N., Wangchuk J., Kondabagil K., Mukherji S. LSPR based fiber optic sensor for detection of *E. coli* using bacteriophage T4. Proceedings of the 2015 Workshop on Recent Advances in Photonics (WRAP).

[109] Gowri A., Sai V. U-bent plastic optical fiber based plasmonic biosensor for nucleic acid detection. Proceedings of the Optical Sensors 2017.

[110] Kumar S., Singh R., Yang Q., Cheng S., Zhang B., Kaushik B.K. (2021). Highly Sensitive, Selective and Portable Sensor Probe Using Germanium-Doped Photosensitive Optical Fiber for Ascorbic Acid Detection. IEEE Sens. J..

[111] Wang Y., Singh R., Chaudhary S., Zhang B., Kumar S. (2022). 2-D Nanomaterials Assisted LSPR MPM Optical Fiber Sensor Probe for Cardiac Troponin I Detection. IEEE Trans. Instrum. Meas..

[112] Kumar S., Singh R., Kaushik B.K., Chen N.-K., Yang Q.S., Zhang X. (2019). LSPR-Based Cholesterol Biosensor Using Hollow Core Fiber Structure. IEEE Sens. J..

[113] Vaiano P., Carotenuto B., Pisco M., Ricciardi A., Quero G., Consales M., Crescitelli A., Esposito E., Cusano A.J.L., Reviews P. (2016). Lab on Fiber Technology for biological sensing applications. Laser Photonics Rev..

[114] Ricciardi A., Crescitelli A., Vaiano P., Quero G., Consales M., Pisco M., Esposito E., Cusano A.J.A. (2015). Lab-on-fiber technology: A new vision for chemical and biological sensing. Analyst.

[115] Liu Q., Liu Y., Yuan H., Wang J., Guang J., Peng W. Smartphone based LSPR biosensor. Proceedings of the Asia Communications and Photonics Conference.

